# Not Quite Folded: Challenges in Predicting the hNPS‐hNPSR‐Ile107 Complex With AlphaFold2 Multimer

**DOI:** 10.1002/cmdc.70449

**Published:** 2026-08-25

**Authors:** Valentina Albanese, Michela Argentieri, Federica Agosta, Alessandra Rizzo, Delia Preti, Rainer K. Reinscheid, Girolamo Calò, Remo Guerrini, Alfonso Carotenuto, Salvatore Pacifico, Chiara Ruzza, Antonella Ciancetta

**Affiliations:** ^1^ Department of Chemical Pharmaceutical and Agricultural Sciences (DOCPAS) University of Ferrara Ferrara Italy; ^2^ Department of Neuroscience and Rehabilitation Laboratory for Technologies of Advanced Therapies (LTTA) University of Ferrara Ferrara Italy; ^3^ Institute of Pharmacology and Toxicology Jena University Hospital Jena Germany; ^4^ Section of Pharmacology Department of Pharmaceutical and Pharmacological Sciences University of Padova Padova Italy; ^5^ Department of Pharmacy University of Naples Federico II Napoli Italy

**Keywords:** AlphaFold2, cyclic peptides, G protein‐coupled receptor, molecular dynamics, neuropeptide S receptor

## Abstract

The human neuropeptide S (NPS) receptor (NPSR) is a Class A peptide G protein‐coupled receptor expressed in the central nervous system and endogenously activated by NPS, a 20‐mer peptide. NPSR activation promotes cellular excitability via Gq and Gs signalling. Studies suggest that receptor antagonists may reduce drug‐seeking behaviours, whilst agonists represent innovative non‐sedating anxiolytics with memory‐enhancing effects. Despite its therapeutic potential, NPSR remains poorly characterised, with neither experimental receptor structures nor drug‐like clinical candidates available. To fill this gap, we applied a previously validated AlphaFold2 Multimer‐based protocol to model the hNPS–hNPSR complex. The model showing higher stability in molecular dynamics simulations and consistency with known structure–activity relationships served as template to design novel hNPS analogues. However, experimental validation through synthesis and in vitro pharmacological evaluation of 20 novel truncated cyclic peptides revealed the model's inability to capture hNPS bioactive conformation, as most analogues were inactive as agonists. By exploiting the stereochemical switch in hNPS hinge region, we identified four novel cyclic antagonists (**17**–**20**, pA_2_ in the 6.10–6.20 range). Our findings highlight strengths and limitations of current peptide‐GPCR modelling strategies and underscore the need for integrating AI predictions with experimental refinement to advance ligand discovery for challenging targets like NPSR.

## Graphical abstract

AlphaFold2 Multimer guided the design of 20 novel human neuropeptide S (hNPS) cyclic analogues. Modelling bias toward the inactive state favoured receptor blockade over activation. Exploiting a stereochemical switch in the peptide hinge region yielded four potent NPS receptor (NPSR) antagonists with pA_2_ up to 6.20. Lines indicate conformational bridges within the hNPS(1–10) sequence.

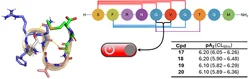

## 1 Introduction

**FIGURE 1:**
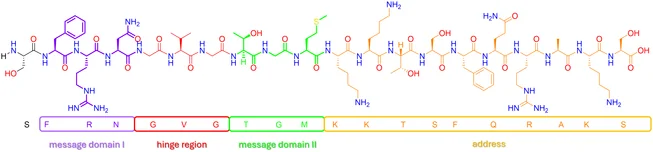
NPS sequence in humans. The four distinct functional domains are highlighted as follows: Ser‐1, Primary Message, Hinge, Secondary Message and Address domains are coloured in grey, purple, red, green and yellow, respectively.

G protein‐coupled receptors (GPCRs) are the largest family of membrane proteins, counting >800 members in humans [1], regulating a plethora of biological functions and representing the target of ~25% of drugs in the clinic [2, 3]. Class A GPCRs, the most numerous GPCR subfamily, comprise validated targets for the treatment of cancer as well as diseases affecting the central nervous system (CNS), the cardiovascular, gastro‐intestinal, and respiratory systems. About 30% of Class A GCPRs are activated by endogenous peptides [4, 5]. Among these, neuropeptide S (NPS) is a 20‐amino acid peptide (human primary sequence: SFRNGVGTGMKKTSFQRAKS) activating the formerly orphan GPCR154, now termed neuropeptide S receptor (NPSR) [6, 7]. Owing its name to the presence of a serine residue at the 1‐position, conserved in all species [7], NPS sequence (Figure 1) is highly conserved among vertebrates and functionally divided into four domains: a primary message (FRN), a hinge region (GVG), a secondary message (TGM) and an address domain (KKTSFQRAKS). Interestingly, the N‐terminal SFRNGVG motif (corresponding to the primary message and the hinge region) is identical in almost all species, with the exception of certain sea turtles (*Caretta caretta*, NCBI accession XP_074987255.1; *Lepidochelys olivacea*, GenBank CAM4591570.1, *Lepidochelys kempii*, NCBI accession XP_073211053.1) and pinnipeds (*Halichoerus grypus*, NCBI accession XP_077933028.1; *Phoca vitulina*, NCBI accession XP_032278908.1) where the SFCNGVG variant has been recently detected [8] and likely represents the overall peptide ‘message’ [9], whereas a few variations in the secondary message domain and at the C‐terminus are observed.

NPSR shows moderate homology to other GPCR family members, the closest relatives being the vasopressin and oxytocin receptors [10]. Receptor activation leads to intracellular Ca^2+^ mobilisation and cAMP formation, therefore promoting cellular excitability via Gq and Gs signalling, respectively [11]. Among known multiple single‐nucleotide polymorphisms (SNP) and splice variants in the human gene, the Asn‐Ile exchange at position 107 (rs324981, A > T substitution) is considered a gain‐of‐function variant: Indeed, hNPSR‐I107 displays similar binding affinity but ~10‐fold higher NPS potency than hNPSR‐N107 [11]. NPS expression is localised to a few brain areas, whereas NPSR receptors are widely distributed in the CNS, in regions associated with stress response, memory and arousal regulation [12]. Central NPS administration in rodents induces arousal and wakefulness, reduces fear and anxiety, promotes learning and memory consolidation, produces analgesia and modulates drug‐seeking behaviours [13]. Hence, NPSR selective agonists represent innovative nonsedating anxiolytics with memory enhancement effects as well as analgesic agents, while NPSR antagonists hold the potential to treat relapse to substances of abuse [14, 15, 16, 17]. Human genetic studies have associated NPSR variants with anxiety and panic disorders, fear sensitivity, stress responsiveness, and alterations in sleep duration [12, 14, 18]. Notably, the anxiolytic effects seem mediated by Gq‐ rather than Gs‐mediated signalling, thus suggesting that Gq‐biased NPSR agonist design might represent a promising strategy for innovative CNS drugs development [19, 20, 21].

Despite the interest and the intense drug discovery efforts conducted over the past two decades, NPSR therapeutic potential has not yet been fully explored due to the lack of high‐affinity drug‐like ligands. To date, no small‐molecule NPSR agonist is known, whereas none of the reported NPSR antagonists [22] has progressed beyond the preclinical stage. There is therefore a compelling need for the development of tool compounds and specifically (small‐molecule) NPSR agonists that can help elucidate uncharted aspects of the NPS/NPSR system and drug‐like antagonists that can contribute to its further validation as target. Progress in agonist development mostly focused on elucidating the structure–activity relationship (SAR) of the endogenous peptide: This led to the identification of NPS sequence alterations achieving antagonism [23]; and of truncated analogues selectively triggering Gq‐signalling cascade, such as NPS(1–10) [24] and the SFKN‐NH_2_ tetrapeptide [20, 25]. Interestingly, the degree of signalling bias seems to correlate with the sequence length. As such, truncated peptides are instrumental for the identification of selective agonists with desired signalling preferences. The development of these molecular tools is grounded in a detailed mapping of the structural determinants required for receptor binding and activation. In particular, SAR data on the full peptide sequence suggest that:

1. Position 2 favours bulky hydrophobic or aromatic residues, as analogues bearing aliphatic side chains such as [Cha^2^]hNPS or [Leu^2^]hNPS retain full agonist activity and efficacy, *para*‐substituted Phenylalanine analogues such as [*p*‐Cl‐Phe^2^]hNPS show enhanced potency, whereas polar (e.g., [Asn^2^]hNPS), charged (e.g., [Asp^2^]hNPS), or bulky and geometrically modified aromatic systems (e.g., two phenyl rings such as in [Dip^2^]hNPS) result in a total loss of receptor activation [26].
2. At position 3, a basic residue is optimal for high potency: its substitution with small hydrophobic chains like in [Nva^3^]hNPS or [Abu^3^]hNPS leads to a significant 10‐ to 40‐fold reduction in potency, whereas bulkier hydrophobic (e.g., [Tyr^3^]hNPS) or acidic (e.g., [Asp^3^]hNPS) residues are not tolerated [27].
3. The native asparagine residue at position 4 seems pivotal for receptor activation, as even conservative substitutions with small polar uncharged residues like in [Gln^4^]hNPS or [Thr^4^]hNPS result in 10‐ to 50‐fold potency loss despite retaining a full agonist profile with an Emax comparable to the native peptide [27].

The present study was primarily aimed at generating 3D models of the hNPSR‐I107‐hNPS complex that could facilitate the design of truncated rigid analogues, hopefully locking the peptide in its bioactive conformation. Complex generation was based on a customised AlphaFold2 (AF2) multimer V3 protocol, that was previously applied to and validated on another Class A Peptide‐GPCR, namely the Nociceptin‐NOP receptor complex [28]. In the NOP study, the model built using the customised protocol was nearly indistinguishable from the experimental structure that was subsequently solved by a different research group [29], therefore providing a robust methodological foundation for our current approach. In this work, we applied the same protocol to the hNPSR‐I107 isoform. It has to be emphasised that, although the in vitro compound characterisation was performed using the mouse receptor (mNPSR), the hNPSR‐I107 isoform shares overall high sequence identity (89.22%) and similarity (93.53%) with mNPSR, that reaches 95% identity in the putative orthosteric site (POS).

## 2 Results and Discussion

### 2.1 Model Generation

**FIGURE 2:**
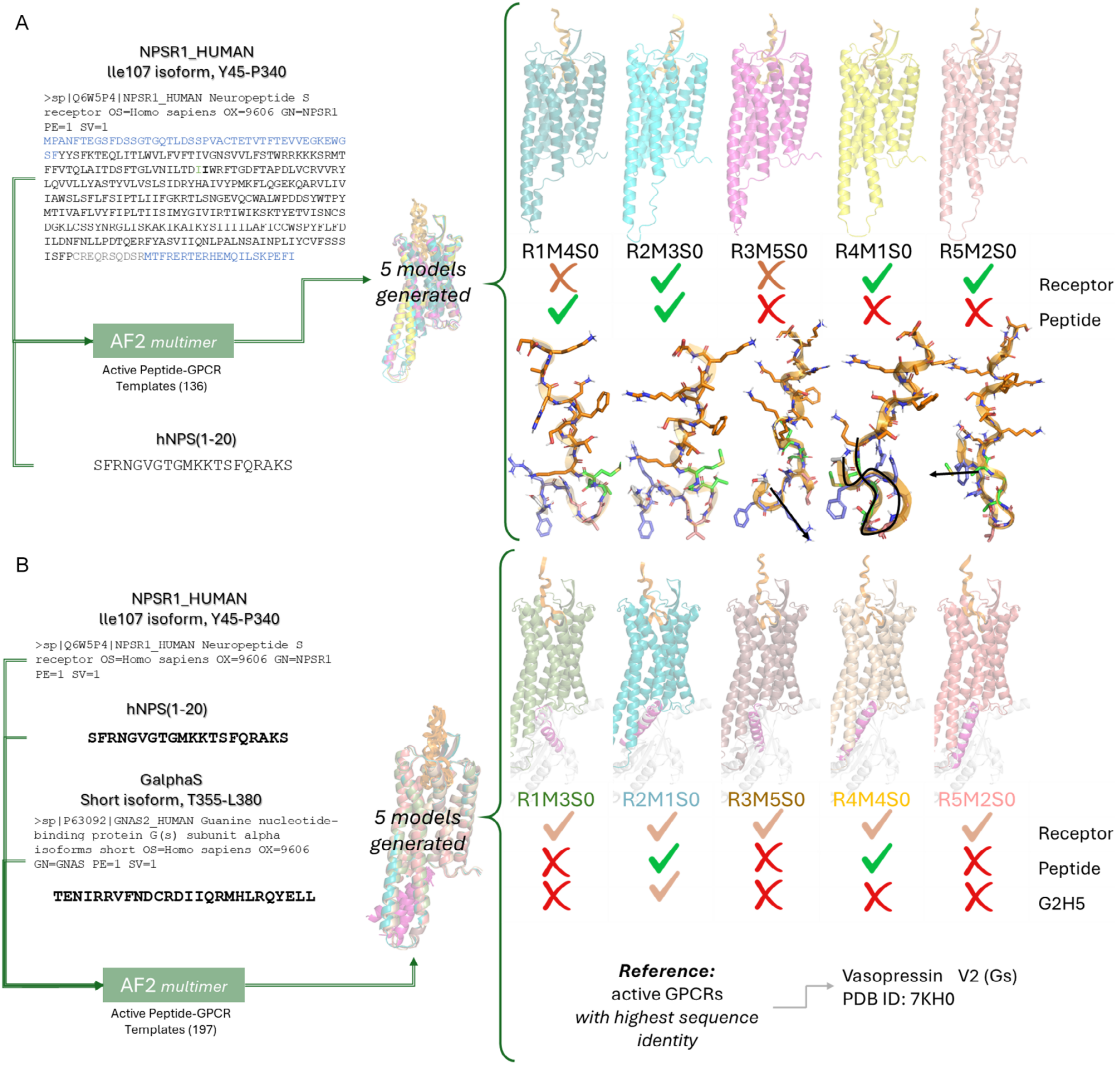
AF2 multimer inputs used to generate (A) binary and (B) ternary NPS‐hNPSRI107/‐GsH5 complexes.

Two sets of AI‐driven models of hNPSR‐I107 in complex with the full length endogenous agonist hNPS(1–20) (binary complexes) and the C‐terminal helix (H5) of the alpha subunit of the Gs protein (hereby denoted as GsH5, pseudo‐ternary complexes) were generated using a previously validated protocol [28] based on AF2 multimer (v3), by feeding the algorithm with the peptide and protein(s) sequences along with two custom template databases of 136 (binary models) and 197 (ternary models) active‐state peptide GPCRs (Left panels in Figure 2A,B, full calculation details and list of templates in the Supporting Information). In all our models, I107^2.63^ is located at the extracellular end of TM2, facing the lipid‐membrane environment and does not appear to be in direct contact with the POS. Although the hNPSR‐Ile107 isoform is a well‐documented gain‐of‐function variant that enhances NPS potency by approximately 10‐fold, the precise molecular mechanism for this effect remains largely unknown. Based on its orientation, we speculate it may influence receptor activity indirectly.

#### 2.1.1 Receptor State and Complex Selection

The five binary complexes (Figure 2A right panel) were carefully inspected and classified based on their structural features. In particular, we focused on the receptor state with the aim at selecting a conformation resembling as much as possible an active‐like state, by taking into account that the intracellular effector was not modelled. Based on the position of the Transmembrane helix 6 (TM6) with respect to the TM bundle, R1M4S0 and R3M5S0 (teal and magenta ribbons in Figure 2A, respectively) were labelled as ‘inactive’ (orange cross) as they featured an inward‐oriented TM6. Models R2M3S0, R4M1S0 and R5M2S0 (cyan, yellow and pink ribbons in Figure 2A, respectively) were labelled as ‘active‐like’, as they featured an outward‐oriented TM6. Interestingly, in both inactive models (R1M4S0 and R3M5S0) the third intracellular loop (ICL3) connecting TM5 to TM6 was partially structured as an alpha helix; in model R2M3S0, TM6 N‐terminus was partially unfolded; whereas in R4M1S0 and R5M2S0, TM6 folded into a full helix. Overall, these models showed AF2 intrinsic struggle to predict TM6 folding, due to low sequence similarity between hNPSR TM6 sequence and the sequences of active GPCRs structures provided as templates. The ternary models (Figure 2B, right panel) featured intermediate receptor conformations (indicated with orange flags) and differed for both the peptide conformation and GsH5 orientation. Of the two models with NPS engaged in reasonable interaction patterns (indicated with green flags), only one model (i.e., R2M1S0), featured a GsH5 orientation (orange flag) resembling the one observed in the X‐ray structure of the V_2_R (PDB ID: 7KH0) in complex with Gs, chosen as reference.

#### 2.1.2 Model Prioritisation and MD Refinement

Based on this preliminary structural analysis, we inspected the peptide orientation and conformation in the POS in each complex. Models R1M4S0 (‘inactive’ receptor conformation) and R2M3S0 (‘active‐like’ receptor conformation) were prioritised for subsequent molecular dynamics (MD) refinement, as they featured the peptide in favourable conformation and engaging in interaction patterns in agreement with known SAR data. Other models were de‐prioritised for structural flaws such as Arg^3^ projected toward the inner TM bundle (R3M5S0, black downward‐directed arrow in Figure 2A, right panel), a severely strained peptide conformation (R4M1S0, tangled black line in Figure 2A, right panel), steric clashes between peptide and receptor residue side‐chains (R5M2S0 black left‐directed arrow in Figure 2A, right panel).

As far as the pseudo‐ternary complexes are concerned, only R2M1S0 and R4M4S0 featured the peptide engaged in reasonable interaction patterns (indicated with green flags in Figure 2B, right panel). Of these two models, R2M1S0 featuring a GsH5 V_2_R‐like orientation, was selected for MD refinement (50 ns, in triplicate with the systems embedded in a solvated phospholipid bilayer).

**FIGURE 3:**
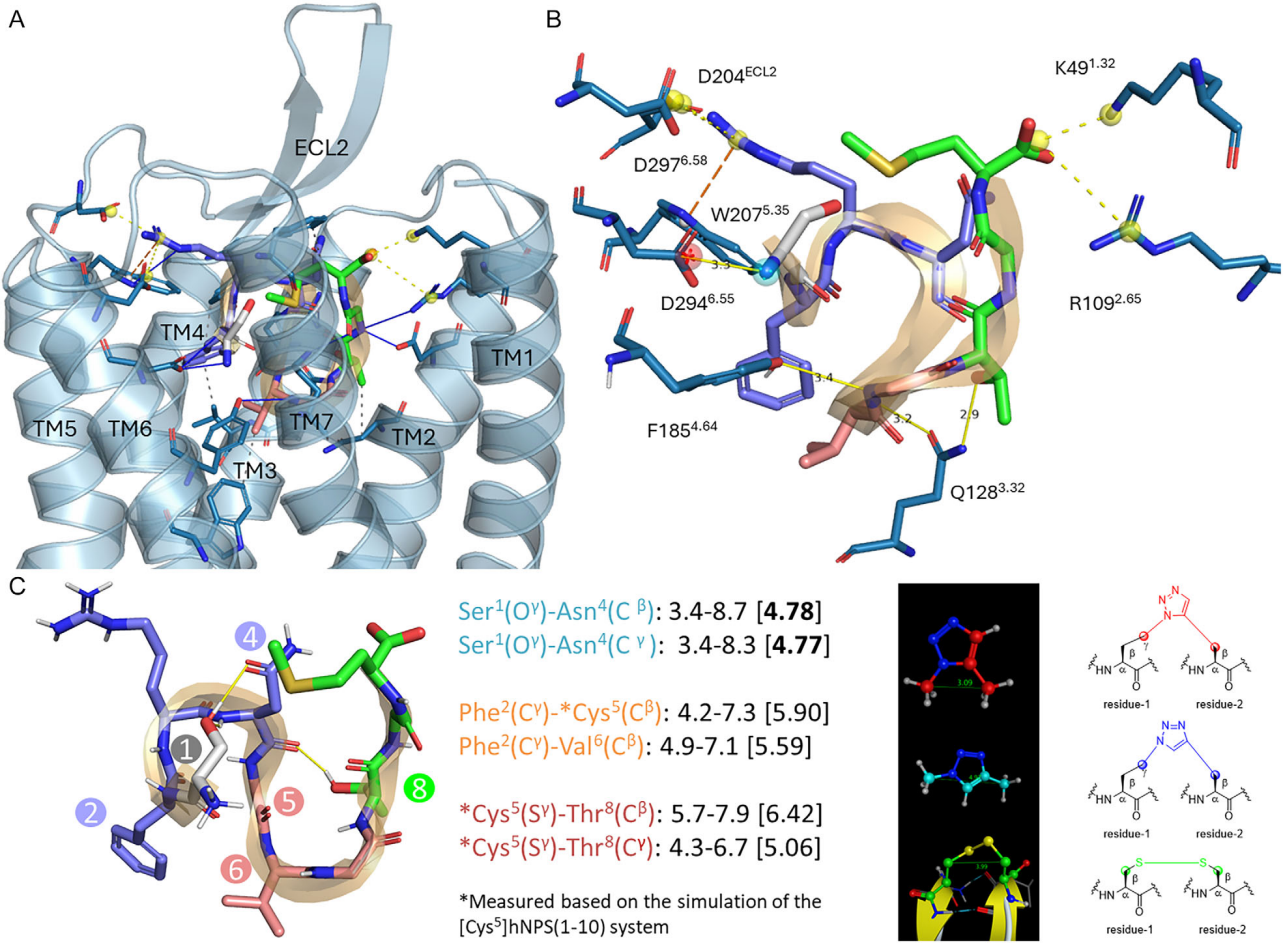
(A) 3D overview of the hNPS(1–10) predicted binding mode within the transmembrane (TM) bundle of the hNPSR: TM1–TM7 helices and the second extracellular loop (ECL2) are labelled. (B) Close‐up view of the key interaction established between hNPS and hNPSR. Receptor carbon atoms are in blue, whereas hNPS carbon atoms are coloured as follows: Ser^1^, grey; Phe^2^‐Arg^3^‐Asn^4^, purple; Gly^5^‐Val^6^‐Gly^7^, red; and Thr^8^‐Gly^9^‐Met^10^, green. Interaction types are represented according to PLIP representation: H‐bonds as blue continuous lines, hydrophobic interactions as grey dashed lines and salt bridges as yellow dashed lines with the charge centre shown as a yellow sphere. (C) Left: Pseudo‐cyclic conformation extracted from R1M4S0 binary model trajectory (run 2) showing potential for cyclic analogue design. Centre: inter‐residue distance range and [average values] in Å calculated between residue side chain pairs (specific atoms in parenthesis). Right: 3D models and 2D chemical structures of the 1,5‐ (red) and 1,4‐disubstituted (blue) 1,2,3‐triazoles and disulphide (green) bridges used to mimic the predicted distances in the truncated cyclic analogue design.

Trajectory analysis (Tables S1 and S2, Figure S1 and Video S1) revealed slight differences between the two selected binary models: In both complexes, the receptor Cα atoms showed comparable stability with average RMSD values between 2.04 and 2.40 Å. The peptide remained stable in the POS ( Video S1) in both complexes, by establishing interactions consistent with known SAR data and showing average RMSD values for hNPS(1–10) sequence around 1.9–2.8 Å. A different behaviour is observed for the peptide address domain hNPS(11–20), showing average RMSD values ≥6.5 Å, indicating the lack of anchoring interactions, presumably ascribed by the fact that the receptor N‐terminus (residues 1–44) was not modelled (See peptide RMSF profiles in Figure S1A,B). Overall, the main TM bundle maintained a stable conformation through the trajectories with the exception of TM5 and TM6 and ICL3, the latter reaching RMSD values as high as 10 Å. Notably, TM5 and TM6 showed higher fluctuations in the inactive conformation (R1M4S0), whereas ICL3 showed the highest fluctuations in the active‐like model (R2M3S0), as expected due to the lack of secondary structure in the initial model. This likely suggests that the partial helix character AF2 imparted on ICL3, although extremely unlikely and unprecedented in class A GPCRs, contributed to the conformational stability of the IC region. Interestingly, the inclusion of GsH5 in the ternary complex (Figure S1C), contributed to stabilise TM5 and TM6 but not ICL3, despite this region folded with a spatial arrangement typical of active state class A GPCRs. To overcome the high conformational flexibility observed in the address domain (residues 11–20) of the full‐length peptide, we modelled the hNPS(1–10) truncated sequence within the R1M4S0 model (showing the lowest average RMSF values for the Cα atoms of the NPS(1–10) sequence) to obtain a more stable binding mode. As shown in Figure S1D and detailed in Table S1, the truncated peptide system showed average RMSD values for the TMs comparable to the full length peptide system. We extracted the energy minimum of the trajectory exhibiting the lowest average peptide Cα atoms RMSD value (run2) and inspected in detail the peptide‐protein interactions. As shown in Figure 3A,B and Video S2, the peptide is deeply buried within the POS, with the N‐terminus pointing towards TM5/TM6 and the C‐terminus facing the TM1/TM2 interface, respectively. The scaffold is anchored by two salt‐bridges: one between the peptide charged N‐terminus and D294^6.55^ side chain and the other between the peptide charged C‐terminus and K49^1.32^ and R109 ^2.65^ side chains. Phe^2^ is hosted in a hydrophobic pocket defined by V129^3.33^ and F185^4.64^. A tight interaction network involving Arg^3^ keeps the scaffold further anchored via salt bridges with D204^ECL2^ and D297^6.58^, and a *π*‐cation interaction with W207^5.35^, on one side, whereas Thr^8^ side chain held the scaffold in the POS via a hydrogen bond with the Q128^3.32^ side chain. Overall, the peptide adopts a pseudo‐cyclic conformation (Figure 3C) stabilised by an intramolecular hydrogen bond between Ser^1^ and Asn^4^ side chains, which is in line with literature SAR data emphasising the pivotal role of the residue side chain at position 4.

The conformational propensity of hNPS(1–10) was supported by a limited number of NOE contacts between the side‐chain protons of residues Phe^2^ and Val^6^ in the NOESY spectrum acquired in the presence of dodecylphosphocholine (DPC) micelles (Figure S2). Thus, the conformational ensemble sampled by receptor‐free hNPS(1–10) appears to include conformers compatible with the modelled receptor‐bound active conformation, suggesting that the bioactive geometry may already be populated in the membrane mimetic environment. Accordingly, receptor binding may occur, at least in part, through a conformational‐selection mechanism whereby the receptor preferentially recognises and stabilises a pre‐existing agonist‐competent subpopulation.

### 2.2 Cyclic Peptide Design and Synthesis

Prompted by the alignment of the predicted binding mode (Figure 3B) to literature SAR data [26, 27], we measured the distances between side chain pairs (Figure 3C) in order to identify cyclisation points that could ‘lock’ the peptide into the aforementioned putative bioactive conformation. To this aim, we carried out an additional simulation of the [Cys^5^]hNPS(1–10) system (see Supporting Information), in order to explore cyclisation strategies involving also position 5. Indeed, this residue represents a critical pharmacological switch [30, 31]: While the native residue (glycine) lacks a side chain, its substitution with L‐configured amino acids, such as in [Val^5^]hNPS or [Cys^5^]hNPS, generally maintains agonist activity, whereas D‐configured residues, such as in [D‐Val^5^]hNPS or [^*t*^Bu‐D‐Gly^5^]NPS, result in full antagonists. Notably, the resulting fold of the mutant system (Figure S3 and Video S3) was superimposable on the native hNPS(1–10) trajectory.

Overall, we designed 20 truncated cyclic analogues (Tables 1 and 2) by pursuing three main cyclisation strategies, namely the introduction of: (i) 1,5‐disubstituted and (ii) 1,4‐disubstituted 1,2,3‐triazoles, as well as (iii) classical disulphide bridges. These options were selected also based on measured inter‐residue distances and in particular the distance between the γ atom of the first residue to the *β* atom of the second residue (see 2D scheme in Figure 3C). As such, 1,5‐ and 1,4‐disubstituted 1,2,3‐triazoles were designed to mimic an inter‐residue distance of 3 and 5 Å, respectively, whereas Cys–Cys disulphides were designed to keep an intermediate inter‐residue distance of 4 Å. A systematic scan was also carried out by evaluating residue pairs from positions 1,4 to 1,7 and from 2,5 to 2,7, along with the additional 5,8 cyclisation, based on the spatial orientations and distances predicted by our models. Additional variations incorporating Homocysteine (HCys) and Penicillamine (Pen) were also explored to optimise the fit within the binding site and ensure all possible distance ranges were covered in the series. D‐Penicillamine (D‐Pen) was also included in the cyclisation scan involving residues in the hinge region to investigate the structural requirements necessary to lock the peptide into an antagonist conformation, based on the abovementioned literature data [30, 31], whilst providing a versatile thiol‐bearing scaffold for further optimisation.

**TABLE 1:** In vitro pharmacological activity of 1–16 analogues and the reference agonist hNPS.

Cpd	Structure	Agonism		Antagonism
		pEC_50_ (CL_95%_)	*E*_max_ (±SEM %)	pA_2_ (CL_95%_)
**hNPS**	Ser‐Phe‐Arg‐Asn‐Gly‐Val‐Gly‐Thr‐Gly‐Met‐OH	**8.75** (8.64–8.86)	**350** (±7%)	—
**1**	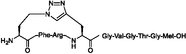	Inactive		<5
**2**	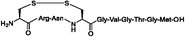	Inactive		<5
**3**	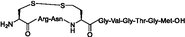	Inactive		<5
**4**	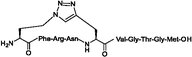	Inactive		<5
**5**	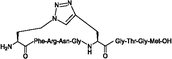	10 μM: 113% ± 9%		<6
**6**	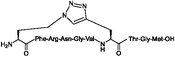	10 μM: 61% ± 7%		<6
**7**	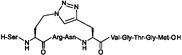	10 μM: 29% ± 4%		<6
**8**	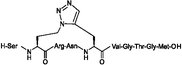	Inactive		<5
**9**	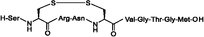	Inactive		<5
**10**	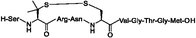	Inactive		<5
**11**	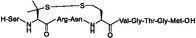	Inactive		<5
**12**	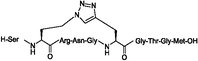	10 μM: 51% ± 16%		<6
**13**	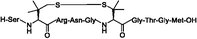	Inactive		<5
**14**	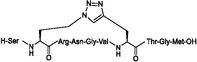	Inactive		<5
**15**	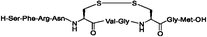	Inactive		<5
**16**	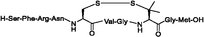	Inactive		5.80 (5.51–6.09)

*Note:* Bold indicates the Compound numbers, and Ic50 values for the reference peptide hNPS.

*Note:* pEC_50_ and pA_2_ values are expressed as mean (CL_95__%_); *E*_max_ values are expressed as mean ± SEM; *N* = 5 experiments performed in duplicate. Inactive indicates no agonist activity up to 10 μM. Compounds inactive as agonists up to 10 μM were tested for antagonism at 10 μM, whereas compounds displaying agonist activity at 10 μM were evaluated for antagonism at 1 μM, corresponding to the highest concentration at which they were inactive per se.

**TABLE 2:** In vitro pharmacological activity of 17–20 analogues and the reference agonist hNPS.

Cpd	Structure	Agonism		Antagonism
		pEC_50_ (CL_95%_)	*E*_max_ (± SEM %)	pA_2_ (CL_95%_)
**hNPS**	Ser‐Phe‐Arg‐Asn‐Gly‐Val‐Gly‐Thr‐Gly‐Met‐OH	**8.5** (8.05–8.88)	**170** (± 30%)	
**17**	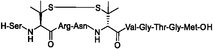	Inactive		6.16 (6.06–6.26)
**18**	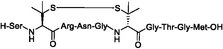	Inactive		6.19 (5.90–6.48)
**19**	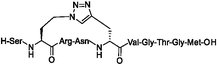	Inactive		6.05 (5.81–6.29)
**20**	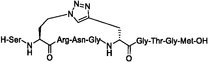	Inactive		6.12 (5.88–6.36)

*Note:* Bold indicates the Compound numbers, and Ic50 values for the reference peptide hNPS.

*Note:* pEC_50_ and pA_2_ values are expressed as mean (CL_95__%_); *E*_max_ values are expressed as mean ± SEM; *N* = 5 experiments performed in duplicate. Inactive indicates no agonist activity up to 10 μM. Compounds were tested for antagonism at 10 μM.

### 2.3 In Vitro Pharmacological Characterisation

**FIGURE 4:**
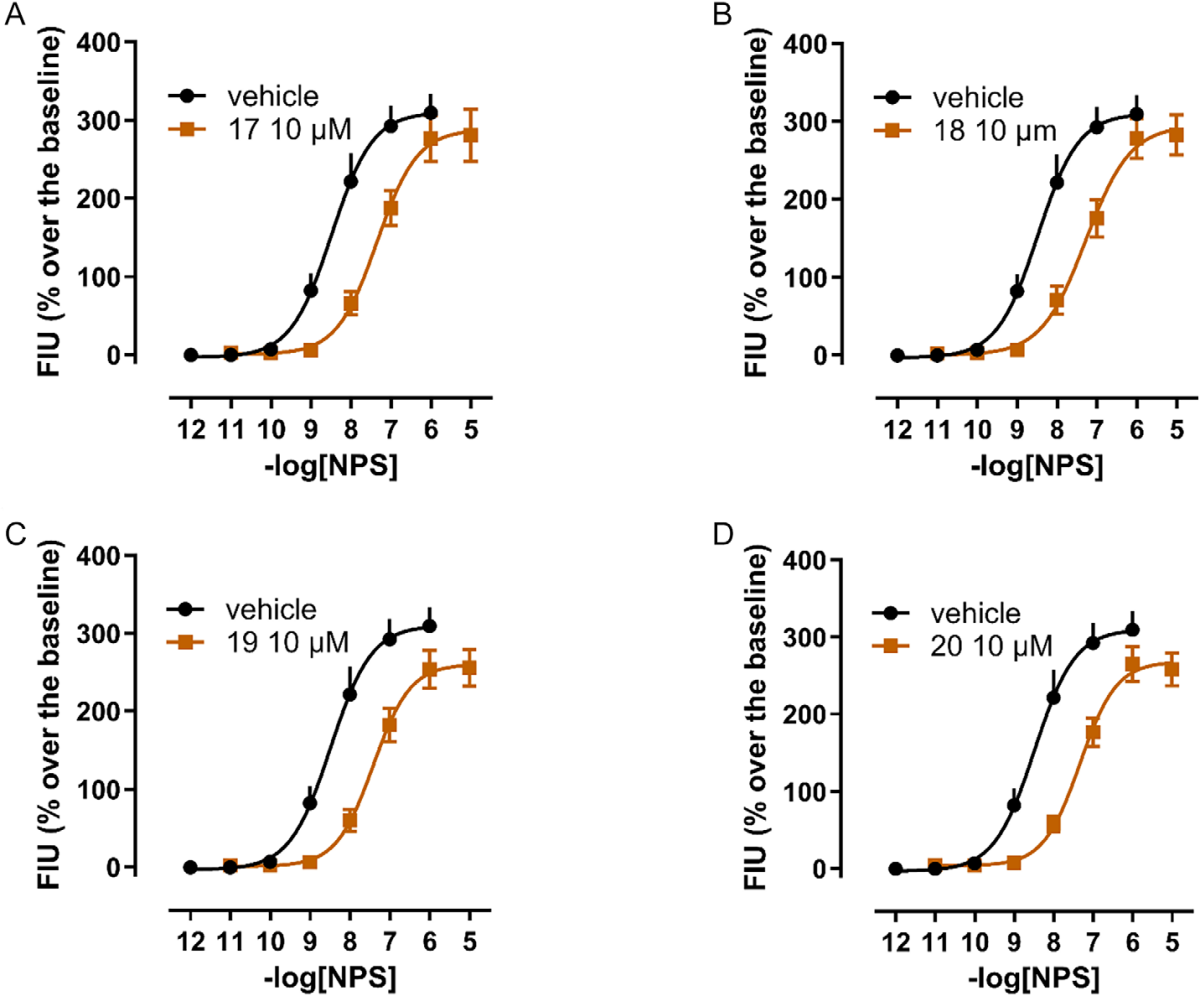
Calcium mobilisation experiments. Concentration–response curve to NPS in the absence (vehicle) and in the presence of 10 µM of **compound 17** (A), **compound 18** (B), **compound 19** (C), and **compound 20** (D). Data are the mean ± SEM of five experiments performed in duplicate.

The pharmacological profile of newly synthetised compounds was evaluated in vitro using a calcium mobilisation assay in HEK293 cells stably expressing mNPSR and results compared to the native **hNPS** profile. As detailed in the experimental section, all compounds were tested as both agonists and antagonists. Compounds inactive up to 10 μM were tested as antagonists at 10 μM, whereas those showing activity at 10 μM were tested as antagonists at 1 μM. As reported in Tables 1 and 2, the cyclisation strategies pursued led to a total loss of agonist activity, with the majority of the compounds resulting inactive up to 10 μM. However, the incorporation of D‐residues in the hinge region to induce antagonism proved successful, as four analogues (**17**–**20**) behaved as full antagonists, effectively showing no agonist activity but shifting the NPS concentration–response curve (Figure 4). In particular, the disulphide‐bridged compounds **17** and **18**, incorporating Pen^2^ and D‐Pen^5/6^, exhibited a pA_2_ = 6.20, whereas triazole‐linked compounds **19** and **20** were slightly less potent (pA_2_ = 6.10). These results demonstrate that, while the introduced conformational constraints are incompatible with receptor activation, they provide a viable scaffold for the development of novel cyclic NPSR antagonists.

## 3 Conclusion

Our study serves as a compelling reminder of a recurring irony in GPCR structure‐based drug design: The most reliable way to discover novel antagonists is often a carefully planned attempt at designing agonists! It also emphasises the current limitations of AI‐driven models: Despite employing a previously validated protocol that proved successful in another peptide‐GPCR case study, and confirming the structural stability of the resulting complex through classical MD, the experimental validation served as a humbling dose of reality. Indeed, the synthesis and in vitro pharmacological evaluation of newly designed 20 truncated cyclic analogues revealed that our model, despite its apparent agreement with established SAR data, failed to capture the bioactive conformation required for NPSR activation. A primary source of error in our approach likely stems from the receptor model representing an inactive state, which, while structurally stable, might be functionally biased toward antagonism and therefore better accommodating antagonist templates. To overcome this limitation, more sophisticated modelling attempts are currently underway in our laboratory, incorporating the entire Gq and Gs alpha subunits in the pseudo‐ternary models, in order to stabilise the active receptor state and provide a better template for agonist design.

The pharmacological results provided in this study confirmed that the conformational constraints designed to ‘lock’ the putative bioactive conformation resulted in the loss of both agonist potency and efficacy. However, the ‘failure’ was strategically redeemed by exploiting the established pharmacological switch at position 5, whereby the introduction of D‐configured residues like D‐penicillamine (D‐Pen) transitions the peptide from agonism to antagonism. Indeed, by introducing D‐amino acids in the hinge region (position 5 and 6) we successfully identified four novel cyclic antagonists (compounds **17**–**20**) with pA_2_ values reaching 6.20.

Ultimately, while AlphaFold2 Multimer provided a structurally stable ‘folded’ hypothesis, the experimental validation through constrained analogue design, synthesis and pharmacological evaluation revealed an unexpected outcome. This discrepancy likely stems from the receptor model representing an inactive conformation, which functionally biased the structure‐based design process toward receptor blockade rather than activation. As such, current and future modelling efforts will incorporate the G protein alpha subunit to better represent and hopefully stabilise the receptor active state. Nonetheless, our work adds a valuable new set of conformationally constrained antagonists to the NPSR toolbox, although the “key” to the agonist state of this elusive drug target is yet to be found.

## 4 Materials and Methods

### 4.1 In Silico Model Generation

A set of five binary models was generated by modelling the Y45‐P340 sequence of human NPSR‐Ile107 isoform (hNPSR‐I107, UniProt ID: Q6W5P4) in complex with the full length endogenous agonist NPS (sequence in human: SFRNGVGTGMKKTSFQRAKS), using a previously validated protocol [28] based on AlphaFold2 (AF2) multimer v3 (as implemented in ColabFold (v1.5.0)). In particular, the algorithm was fed with the peptide and protein sequences along with a custom template database of 136 active‐state GPCRs in complex with a peptide agonists (details in the Supporting Information). Attempts to model a longer hNPSR sequence by including both N‐ and C‐Term residues (or portion thereof) resulted in artificial interactions observed either in the model generation stage (e.g., a longer N‐Term resulted in an elongated TM1 alpha‐helix that biased the hNPS peptide conformation) or during subsequent preliminary MD (e.g., the C‐Term helix 8 interacted with ICL3 and destabilised both TM5 and TM6). A set of pseudo‐ternary complexes was built by including the C‐terminal Helix 5 of the alpha subunit of the Gs protein (hereby denoted as GsH5) and specifically the TENIRRVFNDCRDIIQRMHLRQYELL sequence corresponding to residues 355–380 of Alpha‐S1 (GNASs, Alpha‐S‐short, UniProt ID: P63092) and by augmenting the custom template database with small molecule‐bound or apo active‐state peptide GPCRs for a total of 197 structures. Although we are aware that the experimental setting is based on Ca^2+^ measurement mediated by the Gq protein, at the time we generated the models the number of GPCR structures solved in complex with Gq proteins was too small to provide any useful information for the coupling interface. The models were processed with PLIP [32] and visually inspected: two binary and one pseudo‐ternary complexes were prioritised for MD refinement based on both peptide and receptor conformation as well as the observed peptide‐receptor and receptor‐G protein interaction pattern.

### 4.2 Molecular Dynamics

System setup was carried out using CHARMM‐GUI [33] web‐server with hNPSR‐I107 and GsH5 N‐termini acetylated (ACE), methyl‐amidated (CT3) and charged (CTER) hNPSR‐I107 and GsH5 C‐termini, respectively, while standard charged patches (NTER and CTER) were applied to hNPS(1–20) and hNPS(1–10) termini. A disulphide patch was applied to connect hNPSR‐I107 C121 and C197 side chains in order to enforce ECL2‐TM3 disulphide bridge, conserved in almost Class A GPCRs. The complexes were embedded in a 1‐palmitoyl‐2‐oleoyl‐*sn*‐glycero‐3‐ phosphocholine (POPC) lipid bilayer (80 × 80 Å) by exploiting the “Positioning of proteins in membrane” (PPM2.0) [34] functionality to correctly orient the receptor with respect to the membrane. Membrane‐embedded systems were solvated with TIP3P water and Na^+^/Cl^−^ counter‐ions at a 0.15 M concentration were added to keep the systems neutral. 50 ns of MD production were run in triplicate (NPT Ensemble, 310.15 K, 2 fs integration time step) using OpenMM 8.0 [35] using CHARMM36m Force Field [36] with WYF parameters for cation‐*π* interactions [37] and periodic boundary conditions. System equilibration followed a previously optimised protocol for membrane systems [38], comprising two NVT steps followed by four NPAT steps. A full set of equilibrations followed by a production run was employed for each replica, by choosing random seeds for each step. Harmonic restraints on protein heavy atoms and ions, repulsive planar restraints to prevent water diffusion in the lipid bilayer and planar restraints applied to the lipid head‐groups were gradually released. Temperature and pressure coupling were maintained using Langevin dynamics (friction coefficient = 1 ps^−1^) and a semi‐isotropic Monte Carlo barostat (frequency = 100), respectively. Bond lengths involving hydrogen atoms were constrained by the M‐SHAKE algorithm, and long‐range Coulomb interactions were handled using the particle mesh Ewald summation method (PME) with a non‐bonded cutoff distance of 12 Å and a switching distance of 10 Å. All simulations were carried out on a hybrid CPU/GPU local cluster composed of a 24‐core AMD Ryzen Threadripper TR4 3960X CPU equipped with two NVIDIA RTX 3070 GPUs.

RMSD, RMSF and per‐residue NPS RMSF values were calculated by exploiting the align and rms modules available in the MDAnalysis python toolkit [39] by aligning the trajectories on hNPSR‐I107 Cα atoms. Ligand–protein interaction energies were calculated using a tcl/bash script exploiting the NAMD [40] 2.10 *namdenergy* function. RMSD, RMSF and Interaction Energy graphs were generated with Excel and Matplotlib [41].

### 4.3 Solid Phase Synthesis

**SCHEME 1:**
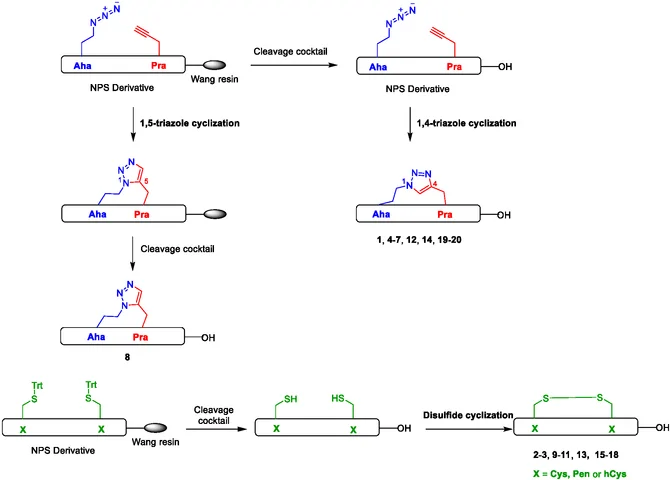
Overview of the cyclisation strategies used in this study.

All linear intermediate peptides were synthesised, as previously described [42], by Fmoc solid phase peptide synthesis (SPPS) on a Wang resin as solid support. The NPS derivatives were obtained by substituting of amino acid residues from native peptide sequence with commercially available building blocks Fmoc‐Cysteine (Fmoc‐Cys(Trt)‐OH), Fmoc‐homoCysteine (Fmoc‐hCys(Trt)‐OH), Fmoc‐Penicillamine (Fmoc‐Pen(Trt)‐OH), Fmoc‐Propargylglycine (Fmoc‐Pra‐OH), and Fmoc‐azidohomoalanine (Fmoc‐Aha‐OH) according to the cyclisation reaction that was intended to be produced (Scheme 1). For the **1, 4**–**7, 12, 14, 19**–**20** and **8** peptides, the native amino acids were replaced by Fmoc‐Pra‐OH and Fmoc‐Aha‐OH. In the **1, 4**–**7, 12, 14** and **19**–**20** cyclic NPS derivative, the cyclisation through a 1,4‐disubstituted 1,2,3‐triazole occurred in solution after cleaving the peptide from the solid support under acidic condition. This cyclisation was performed by CuAAC reaction in water solution with CuSO_4_ as catalyst in the presence of citric acid. Reaction progress was tracked by analytical HPLC, which showed that the retention times of all cyclic structures were shorter than those of the corresponding linear forms. For peptide **8**, the cyclisation via 1,5‐disubstituted 1,2,3‐triazole was achieved on solid support using Cp*RuCl(cod) as the catalyst, microwave irradiation (60 °C) and anhydrous conditions to prevent the decomposition of the ruthenium catalyst. The conversion of the starting material into the macrocyclic peptide **8** was monitored by infrared analysis of the peptide on solid support, focusing on the disappearance of the azide IR absorption band at 2100 cm^−1^. For the disulphide derivatives **2**–**3, 9**–**11, 13** and **15**–**18**, it was necessary to introduce residues with thiol‐bearing side chains (Fmoc‐Cys(Trt)‐OH, Fmoc‐hCys(Trt)‐OH, Fmoc‐Pen(Trt)‐OH) in place of the native amino acids. The cyclisation by a disulphide link was performed under diluted conditions (1 mg/mL) dissolving the linear peptide in a mixture of DMSO/H_2_O/TFA in a 25/75/0.1 ratio. The reaction progress was again monitored by analytical HPLC. All cyclic NPS derivative peptides were purified by reverse‐phase HPLC, yielding purities above 98% and characterised by ESI‐MS (HPLC‐chromatograms and MS‐spectra are available in the Supporting Information).

### 4.4 NMR Analysis

The samples for NMR spectroscopy were prepared by dissolving the appropriate amount of peptide hNPS(1–10) in 0.45 mL ^1^H_2_O (pH 5.5), 0.05 mL ^2^H_2_O to obtain a concentration 1 mM of peptide and 200 mM dodecylphosphocholine‐d_38_ (DPC‐d_38_, Cambridge Isotope Laboratories). NMR spectra were recorded on a Varian INOVA 700 MHz spectrometer equipped with a z‐gradient 5 mm triple‐resonance probe head at a temperature of 25 °C. Nuclear Overhauser enhancement spectroscopy (NOESY) spectrum was recorded in the phase‐sensitive mode. Data block sizes were 2048 addresses in *t*_2_ and 512 equidistant *t*_1_ values. Before Fourier transformation, the time domain data matrices were multiplied by shifted sin2 functions in both dimensions. NOESY experiment was run with mixing times of 100 ms. The employment of DPC micelles to investigate the conformational properties of peptides is justified based on their interaction with a membrane receptor hypothesising a membrane‐assisted mechanism of interactions between the peptides and their receptors [43, 44, 45].

### 4.5 Calcium Mobilisation Assay

HEK293_mNPSR_ cells were maintained in Dulbecco's Medium (DMEM) supplemented with 10% fetal bovine serum, 2 mmol/L l‐glutamine and 100 mg/L hygromycin. The cells were cultured at 37 °C in 5% CO_2_ humidified air and seeded at a density of 50,000 cells/well into poly‐d‐Lysine coated 96‐well black, clear‐bottom plates. The following day, the cells were incubated with a medium supplemented with 2.5 mmol/L probenecid, 3 μmol/L calcium‐sensitive fluorescent dye Fluo‐4 am and 0.01% pluronic acid for 30 min at 37 °C. After that time, the loading solution was aspirated, and 100 μL/well of assay buffer Hank's Balanced Salt Solution (HBSS) supplemented with 20 mmol/L 4‐(2‐hydroxyethyl)‐1‐piperazineethanesulfonic acid (HEPES), 2.5 mmol/L probenecid, and 500 μmol/L Brilliant Black (Sigma‐Aldrich, St. Louis, MO) was added. Concentrated solutions (1 mmol/L) of NPS and cyclic analogues were made in bidistilled water. Serial dilutions were carried out in HBSS/HEPES (20 mmol/L) buffer (containing 0.02% bovine serum albumin (BSA) fraction V). After placing both plates (cell culture and compound plate) into the FlexStation II, fluorescence changes were measured at 37 °C. Online additions were carried out in a volume of 50 μL/well. In antagonist‐type experiments, the compounds under study were preincubated for 24 min before agonist addition. To facilitate drug diffusion into the wells in antagonist‐type experiments, three cycles of mixing (25 μL from each well moved up and down three times) were performed immediately after antagonist injection into the wells. Maximum change in fluorescence, expressed in percent of baseline fluorescence, was used to determine agonist response. Data were expressed as mean ± SEM of at least five independent experiments made in duplicate. Nonlinear regression analysis using GraphPad Prism software (v.10.6.1; GraphPad Software LLC) allowed logistic interactive fitting of the resultant responses and the calculation of agonist potencies and maximal effects. Agonist potencies were given as pEC_50_ (the negative logarithm to base 10 of the molar concentration of an agonist that produces 50% of the maximal possible effect). Antagonist potencies were assayed at a single concentration (1 or 10 µM) against the concentration–response curve to NPS. pA_2_ was derived using the following equation: pA_2_ = log(CR − 1) − log[*A*], where CR is the ratio between agonist potency (EC_50_) in the presence and in the absence of antagonist and [*A*] is the molar concentration of antagonist.

## Funding

This study was supported by Ministero dell'Università e della Ricerca (PRIN 2022FPWYA8, PNRR D.M. 352/2022 – M4C2 I.3.3) and Università degli Studi di Ferrara (FAR 2024).

## Conflicts of Interest

The authors declare no conflicts of interest.

## Supporting information

Details on the AI model generation, full list of templates used, RMSD (Table S1) and RMSF (Table S2) profiles of systems simulated in triplicate, peptide RMSF analysis (Figure S1), NOESY spectrum of hNPS(1–10) (Figure S2), MD analysis of the [Cys^5^]hNPS(1–10) system (Figure S3), trajectory Videos S1–S3, and HPLC‐chromatograms and MS‐spectra of peptides **1**–**20** are available as Supporting Information.

## Data Availability

The data that support the findings of this study are openly available in Zenodo at https://doi.org/10.5281/zenodo.20160459.
