# Supplementary material for: Not Quite Folded: Challenges in Predicting the hNPS‐hNPSR‐Ile107 Complex With AlphaFold2 Multimer
Source: ChemMedChem. 2026 Aug 25;21(16):e70449. doi: 10.1002/cmdc.70449 (PMC13507374; doi:10.1002/cmdc.70449)
Supplement: Supplementary file 1 [file CMDC-21-e70449-s001.pdf]

Supporting Information for

Not Quite Folded: Challenges In Predicting the hNPS-hNPSR-Ile107 Complex with AlphaFold2 Multimer

Valentina Albanese,#[a] Michela Argentieri,#[b] Federica Agosta,[a] Alessandra Rizzo,[a] Delia Preti,[a] Rainer K. Reinscheid,[c] Girolamo Calò,[d] Remo Guerrini,[a] Alfonso Carotenuto,[e] Salvatore Pacifico,*[a] Chiara Ruzza,*[b] and Antonella Ciancetta*[a]

[a] *Department of Chemical, Pharmaceutical and Agricultural Sciences, University of Ferrara, Via Luigi Borsari 46, 44121 Ferrara, Italy.*
[b] *Department of Neuroscience and Rehabilitation, University of Ferrara, Via Luigi Borsari 46, 44121 Ferrara, Italy.*
[c] *Institute of Pharmacology and Toxicology, Jena University Hospital, 07747 Jena, Germany.*
[d] *Department of Pharmaceutical and Pharmacological Sciences, Section of Pharmacology, University of Padova, Largo Meneghetti, 2, 35131 Padova, Italy.*
[e] *Department of Pharmacy, University of Naples Federico II, via Montesano 49, 80131 Napoli, Italy*

# These authors contributed equally to this work

* Corresponding authors
Antonella Ciancetta, Email: cncnnl1@unife.it
Salvatore Pacifico, Email: pcfsvt@unife.it
Chiara Ruzza, Email: rzzchr@unife.it

CONTENTS **Pag.**

AlphaFold2 Settings	*S2*
Template List	*S5*
Table **S1**. Average Cα RMSD values computed during 50 ns of MD	*S6*
Table **S2**. Average Cα RMSF values computed during 50 ns of MD	*S7*
Figure **S1.** Peptide RMSF analysis computed during 50 ns of MD	*S8*
Figure **S2**. NOESY spectrum of hNPS(1-10)	*S9*
Figure **S3**. MD Analysis of the [Cys^5]hNPS(1-10) system	*S10*
HPLC traces of peptides **1**-**20**	*S11-19*
Table **S3**. ESI-MS data (calculated and observed) for the synthesized peptides	*S21*

AlphaFold2 Settings

hNPSR-I107-hNPS(1-20) System

- Modelled Sequence: hNPSR-Ile107(Y45-P340):hNPS(1-20)
  YYSFKTEQLITLWVLFVFTIVGNSVVLFSTWRRKKKSRMTFFVTQLAITDSFTGLVNILTDIIWRFTGDFTAPDLVCRVVRYLQVVLLYASTYVLVSLSIDR
  YHAIVYPMKFLQGEKQARVLIVIAWSLSFLFSIPTLIIFGKRTLSNGEVQCWALWPDDSYWTPYMTIVAFLVYFIPLTIISIMYGIVIRTIWIKSKTYETVI
  SNCSDGKLCSSYNRGLISKAKIKAIKYSIIIILAFICCWSPYFLFDILDNFNLLPDTQERFYASVIIQNLPALNSAINPLIYCVFSSSISFP:SFRNGVGTG
  MKKTSFQRAKS

- AlphaFold Settings: ColabFold v1.5.0: AlphaFold2 using MMseqs2 [AlphaFold-multimer-v3]
  - **num_relax:** 5
  - **template_mode:** custom

  Advanced settings

    - **model_type:** alphafold2_multimer_**v3**
    - **num_recycles:** auto (if auto selected, will use 20 recycles if model_type=alphafold2_multimer_v3 (with tol=0.5))
    - **recycle_early_stop_tolerance:** auto

  Sample settings

  - **max_msa:** auto (enable dropouts and increase number of seeds to sample predictions from uncertainty of the model)
  - **num_seeds:** 1
  - **use_dropout:** NO

- Templates found:
  - hNPSR-Ile107(Y45-P340): ['7wq3_R', '7l1u_R', '7ezm_D', '7ezh_D', '7ezk_D', '7wq4_R', '7ryc_O', '7f8v_R', '7f8w_R', '7p02_R', '7p00_R', '7rmi_R', '7rmg_R', '7rmh_R', '7vgx_R', '8f7x_R', '6dde_R', '6ddf_R', '8f7r_R', '8f7r_M']

- hNPS(1-20) : ['7ki0_A', '7vbh_A', '6e3y_A', '6uun_A', '6uva_A', '6uus_A', '6b3j_A', '6p9x_A', '6b3j_P']

hNPSR-I107-hNPS(1-20)-GsH5 System

- Modelled Sequence: hNPSR-Ile107(Y45-P340):hNPS(1-20):GsH5(355-380)
  YYSFKTEQLITLWVLFVFTIVGNSVVLFSTWRRKKKSRMTFFVTQLAITDSFTGLVNILTDIIWRFTGDFTAPDLVCRVVRYLQVVLLYASTYVLVSLSIDR
  YHAIVYPMKFLQGEKQARVLIVIAWSLSFLFSIPTLIIFGKRTLSNGEVQCWALWPDDSYWTPYMTIVAFLVYFIPLTIISIMYGIVIRTIWIKSKTYETVI
  SNCSDGKLCSSYNRGLISKAKIKAIKYSIIIILAFICCWSPYFLFDILDNFNLLPDTQERFYASVIIQNLPALNSAINPLIYCVFSSSISFP:SFRNGVGTG
  MKKTSFQRAKS: TENIRRVFNDCRDIIQRMHLRQYELL

- AlphaFold Settings: ColabFold v1.5.0: AlphaFold2 using MMseqs2 [AlphaFold-multimer-v3]
  - **num_relax:** 5
  - **template_mode:** custom

  Advanced settings

  - **model_type:** auto
  - **num_recycles:** auto (if auto selected, will use 20 recycles if model_type=alphafold2_multimer_v3 (with tol=0.5))
  - **recycle_early_stop_tolerance:** auto

  Sample settings

  - **max_msa:** auto (enable dropouts and increase number of seeds to sample predictions from uncertainty of the model)
  - **num_seeds:** 1
  - **use_dropout:** NO

- Templates found:

- hNPSR-Ile107(Y45-P340): ['7wq3_R', '7l1u_R', '7ezm_D', '7l1v_R', '7ezh_D', '7ezk_D', '7mbx_R', '7wq4_R', '7ryc_O', '7f8w_R', '7f8v_R', '7mby_R', '7p02_R', '7p00_R', '7rmi_R', '7rmg_R', '7rmh_R', '7vgx_R', '8f7x_R', '6ddf_R']

- hNPS(1-20) : ['6b3j_P', '7wvy_L', '7bb7_H', '7bb6_H', '7tyo_P', '7tyh_P', '7tyn_P', '7tyw_P', '7tyy_P', '7f2o_D']

- GsH5(355-380) : ['7fig_R', '7fih_R', '6m1h_B', '6vn7_R', '6nbi_P', '7fii_R', '6nbf_P', '6nbh_P', '7vv4_L', '8f7q_R']

Template List

Custom 136 templates:

- 4grv, 4xee, 4xes, 4xt1, 4xt3, 5t04, 5wb2, 6b3j, 6dde, 6ddf, 6e3y, 6jod, 6lmk, 6lml, 6m1h, 6nbf, 6nbh, 6nbi, 6niy, 6os0, 6os1, 6os2, 6os9, 6osa, 6p9x, 6pt2, 6pwc, 6up7, 6uun, 6uus, 6uva, 6vcb, 6whc, 6wpw, 6wwz, 7ad3, 7aue, 7bb6, 7bb7, 7cz5, 7d68, 7dw9, 7eib, 7ezh, 7ezk, 7ezm, 7f1q, 7f1r, 7f2o, 7f4i, 7f8v, 7f8w, 7f9y, 7f9z, 7f16, 7f53, 7f54, 7f55, 7fim, 7fin, 7fiy, 7kh0, 7ki0, 7ki1, 7l0p, 7l0q, 7l0r, 7l0s, 7l1u, 7lll, 7lly, 7na7, 7o7f, 7p00, 7p02, 7piu, 7piv, 7rbt, 7rgp, 7rkf, 7rkm, 7rkn, 7rmg, 7rmh, 7rmi, 7rtb, 7ryc, 7s1m, 7s3i, 7s8l, 7s8m, 7t6t, 7t6u, 7t6v, 7t11, 7tyf, 7tyh, 7tyi, 7tyl, 7tyn, 7tyo, 7tyw, 7tyx, 7tyy, 7tzf, 7v9m, 7v35, 7vab, 7vbh, 7vbi, 7vdl, 7vdm, 7vgx, 7vl9, 7vla, 7vuy, 7vuz, 7vv0, 7vv3, 7vv4, 7w0n, 7w0o, 7w0p, 7w2z, 7wq3, 7wq4, 7wvu, 7wvv, 7wvw, 7wvx, 7wvy, 8f7q, 8f7r, 8f7s, 8f7w, 8f7x

Addition to the template database:

- 5unf, 5ung, 5unh, 5uz7, 5xjm, 6b3j, 6b73, 6dde, 6ddf, 6do1, 6e3y, 6jod, 6lfm, 6lfo, 6lmk, 6lml, 6lpb, 6lw5, 6m1h, 6m1i, 6nbf, 6nbh, 6nbi, 6niy, 6omm, 6orv, 6os0, 6os1, 6os2, 6os9, 6osa, 6p9x, 6p9y, 6pb0, 6pb1, 6pt2, 6pt3, 6pwc, 6up7, 6uun, 6uus, 6uva, 6vcb, 6vn7, 6whc, 6wi9, 6wpw, 6wwz, 6wzg, 6x18, 6x19, 6x1a, 6xox, 7ad3, 7aue, 7bb6, 7bb7, 7c2e, 7c6a, 7cz5, 7d3s, 7d68, 7dty, 7duq, 7dur, 7dw9, 7e14, 7e32, 7eib, 7evm, 7ezh, 7ezk, 7ezm, 7f16, 7f1q, 7f1r, 7f1s, 7f2o, 7f4d, 7f4f, 7f4h, 7f4i, 7f53, 7f54, 7f55, 7f58, 7f8v, 7f8w, 7f9y, 7f9z, 7fig, 7fih, 7fii, 7fim, 7fin, 7fiy, 7jni, 7kh0, 7ki0, 7ki1, 7l0p, 7l0q, 7l0r, 7l0s, 7l1u, 7l1v, 7lci, 7lcj, 7lck, 7lll, 7lly, 7mbx, 7mby, 7na7, 7na8, 7o7f, 7p00, 7p02, 7piu, 7piv, 7ra3, 7rbt, 7rg9, 7rgp, 7rkf, 7rkm, 7rkn, 7rmg, 7rmh, 7rmi, 7rtb, 7ryc, 7s1m, 7s3i, 7s8l, 7s8m, 7s8n, 7s8o, 7t10, 7t11, 7t6s, 7t6t, 7t6u, 7t6v, 7tyf, 7tyh, 7tyi, 7tyl, 7tyn, 7tyo, 7tyw, 7tyx, 7tyy, 7tzf, 7v35, 7v9m, 7vab, 7vbh, 7vbi, 7vdh, 7vdl, 7vdm, 7vgx, 7vl8, 7vl9, 7vla, 7vuy, 7vuz, 7vv0, 7vv3, 7vv4, 7vv5, 7vv6

Table S1. Average Cα Root Mean Square Deviation (RMSD), expressed in Ångström (Å), computed during a 50 ns MD trajectory (in triplicate) for the solvated membrane-embedded hNPS/hNPS(1-10)-NPSR receptor complexes.

	hNPSR-I107-hNPS(1-20)						hNPSR-I107-hNPS(1-20)-GsH5			hNPSR-I107-hNPS(1-10)		
	R1M4S0			R2M3S0			R2M1S0			R1M4S0		
	Run1	**Run2**	Run3	Run1	Run2	Run3	Run1	Run2	Run3	Run1	Run2	Run3
Cα NPSR	2.16	**2.04**	2.40	2.40	**2.05**	3.06	2.26	2.25	2.20	2.13	2.33	2.13
Cα NPS	5.12	**4.99**	8.25	6.44	**5.29**	5.75	7.09	5.28	6.78	2.39	**2.26**	3.21
Cα NPS(1-10)	1.99	**1.92**	2.35	**2.28**	2.81	2.61	2.08	2.37	2.43	2.39	**2.26**	3.21
Cα NPS(11-20)	6.95	**6.77**	11.41	8.81	**6.89**	7.68	9.81	7.06	9.26	nan	nan	nan
Cα GsH5	nan	nan	nan	nan	nan	nan	3.14	3.33	2.70	nan	nan	nan
TM1	1.60	**1.47**	2.07	**1.43**	1.76	2.20	1.90	2.15	1.82	2.04	2.24	**1.37**
TM2	1.35	**1.32**	1.61	**1.25**	1.30	1.55	2.30	1.64	1.42	1.64	1.64	**1.29**
TM3	1.27	**1.07**	1.57	1.30	**1.01**	1.35	1.34	1.22	1.28	1.45	**1.36**	1.39
TM4	1.22	**1.20**	1.45	**1.26**	1.29	1.40	1.01	1.27	1.29	1.35	1.29	**1.15**
TM5	**2.28**	2.39	2.81	**1.68**	1.95	2.93	1.64	1.78	1.59	**1.99**	2.59	2.53
TM6	2.17	**1.71**	2.42	2.23	**1.95**	3.35	1.37	2.19	2.12	**1.60**	2.06	1.63
TM7	1.65	**1.20**	2.03	**1.58**	1.76	2.24	2.28	2.21	2.17	2.05	1.54	1.45
ECL1	2.64	**2.40**	3.28	**1.28**	1.64	2.08	3.75	3.42	2.37	2.84	**1.68**	2.40
ECL2	2.78	**2.59**	2.63	**1.65**	2.33	2.43	3.85	3.01	2.85	**2.09**	2.32	2.67
ECL3	2.46	**1.79**	2.22	**2.59**	2.93	2.81	2.72	5.12	5.74	2.86	**2.70**	3.19
ICL1	**2.22**	2.76	3.41	3.55	**2.15**	3.09	2.92	1.81	1.66	**1.75**	2.97	1.85
ICL2	3.81	3.39	**2.59**	2.23	**1.83**	2.90	2.36	2.73	1.89	3.96	**2.23**	3.13
ICL3	**4.75**	5.01	5.18	9.36	**5.58**	10.21	5.06	3.70	4.54	**4.02**	5.67	5.14
H8	3.37	**3.11**	3.13	**3.00**	3.36	5.13	3.07	3.83	3.47	4.13	4.51	**3.36**

Table S2. Average Cα Root Mean Square fluctuation (RMSF) values, expressed in Ångström (Å), computed during a 50 ns MD trajectory (in triplicate) for the solvated membrane-embedded hNPS/hNPS(1-10)-NPSR receptor complexes.

	hNPSR-I107-hNPS(1-20)						hNPSR-I107-hNPS(1-20)-GsH5			hNPSR-I107-hNPS(1-10)		
	R1M4S0			R2M3S0			R2M1S0			R1M4S0		
	Run1	**Run2**	Run3	Run1	Run2	Run3	Run1	Run2	Run3	Run1	Run2	Run3
Cα NPSR	1.36	**1.12**	1.17	1.49	**1.25**	1.29	1.15	1.14	1.28	1.35	**1.17**	1.24
Cα NPS	3.19	**1.78**	4.29	**2.38**	3.08	4.80	2.80	2.39	2.21	1.09	**0.73**	1.31
Cα NPS(1-10)	**0.63**	0.81	0.90	1.27	1.14	**0.95**	1.43	1.18	1.59	1.09	**0.73**	1.31
Cα NPS(11-20)	4.47	**2.36**	5.98	**3.07**	4.18	6.70	3.66	3.14	2.64	nan	nan	nan
Cα GsH5	nan	nan	nan	nan	nan	nan	2.45	2.19	2.31	nan	nan	nan
TM1	1.15	**0.87**	1.00	1.01	**0.81**	0.96	1.17	0.98	1.07	1.18	1.07	**0.98**
TM2	0.92	**0.77**	0.78	0.86	**0.79**	0.82	0.96	0.80	0.98	0.93	0.88	**0.82**
TM3	0.80	**0.69**	0.75	0.80	0.80	**0.78**	0.69	0.70	0.84	0.80	**0.65**	0.75
TM4	0.84	**0.73**	0.84	0.81	**0.80**	0.86	0.70	0.71	0.85	0.84	**0.77**	0.81
TM5	1.52	**1.19**	1.28	1.27	1.20	**1.18**	1.04	1.13	1.15	1.40	**1.15**	1.18
TM6	1.25	1.02	**0.94**	1.49	**1.10**	1.21	0.90	1.08	1.15	1.06	**1.03**	1.16
TM7	1.03	**0.77**	0.96	0.98	**0.84**	0.90	1.01	1.09	1.17	1.08	1.03	**1.02**
ECL1	1.10	**0.90**	1.14	**0.98**	1.14	1.07	1.39	1.39	1.64	1.57	**1.14**	1.28
ECL2	**1.06**	1.31	1.58	**1.11**	1.54	1.49	1.37	1.27	1.59	**1.60**	1.66	1.69
ECL3	1.54	**1.26**	1.42	1.56	1.51	**1.47**	1.42	1.56	1.68	**1.56**	1.58	2.09
ICL1	1.77	**1.62**	1.68	1.51	**1.39**	1.67	1.14	1.18	1.32	1.60	**1.45**	1.54
ICL2	1.72	2.40	**1.62**	1.42	**1.25**	1.82	1.18	1.17	1.50	2.62	**1.24**	2.15
ICL3	3.67	**2.10**	2.11	5.13	**3.59**	3.79	2.09	2.48	2.96	2.92	**2.14**	2.27
H8	**1.58**	1.66	1.81	2.38	1.95	**1.48**	2.44	1.79	1.23	1.98	2.11	**1.74**

Figure S1. Average peptide RMSF (graphs) and Cα RMSD (Tables) values resulting from 50 ns of MD simulations in triplicate. Replicas colour code: run1, blue; run2, orange; run3, green. A) hNPS binary complex R1M4S0; B) hNPS binary complex R2M3S0; C) hNPS ternary complex R2M1S0; D) hNPS(1-10) binary complex R1M4S0.

A)

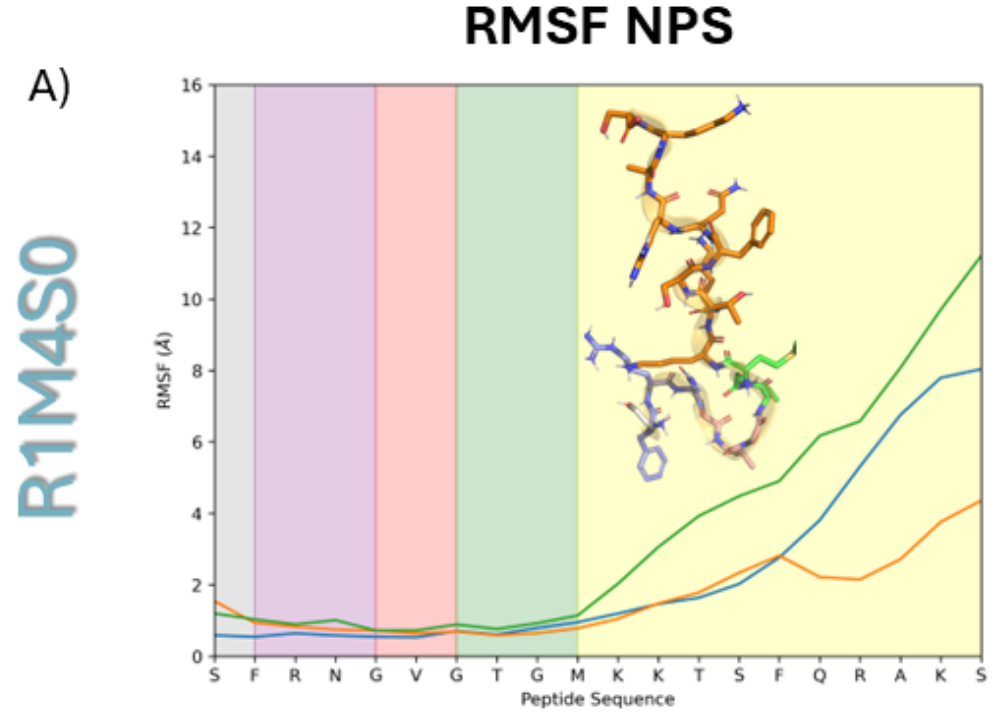

RMSD [Å]

	Cα Receptor	Cα NPS(1-10)
R1	2.16 ± 0.35	1.99 ± 0.27
R2	**2.03 ± 0.36**	**1.92 ± 0.29**
R3	2.40 ± 0.44	2.35 ± 0.30

B)

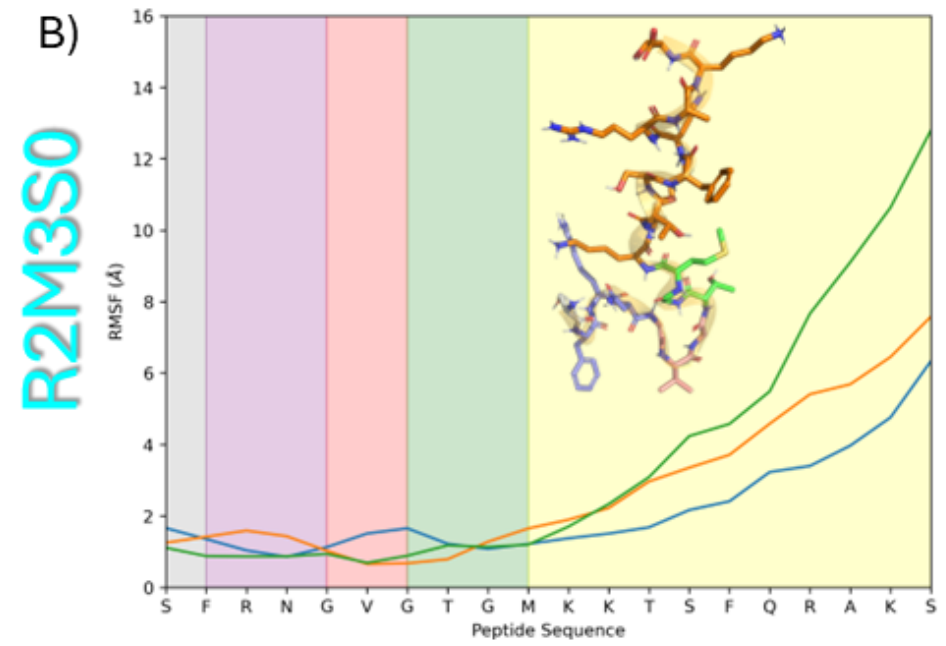

RMSD [Å]

	Cα Receptor	Cα NPS(1-10)
R1	2.40 ± 0.45	**2.27 ± 0.55**
R2	**2.05 ± 0.24**	2.81 ± 0.89
R3	3.06 ± 0.41	2.61 ± 0.40

C)

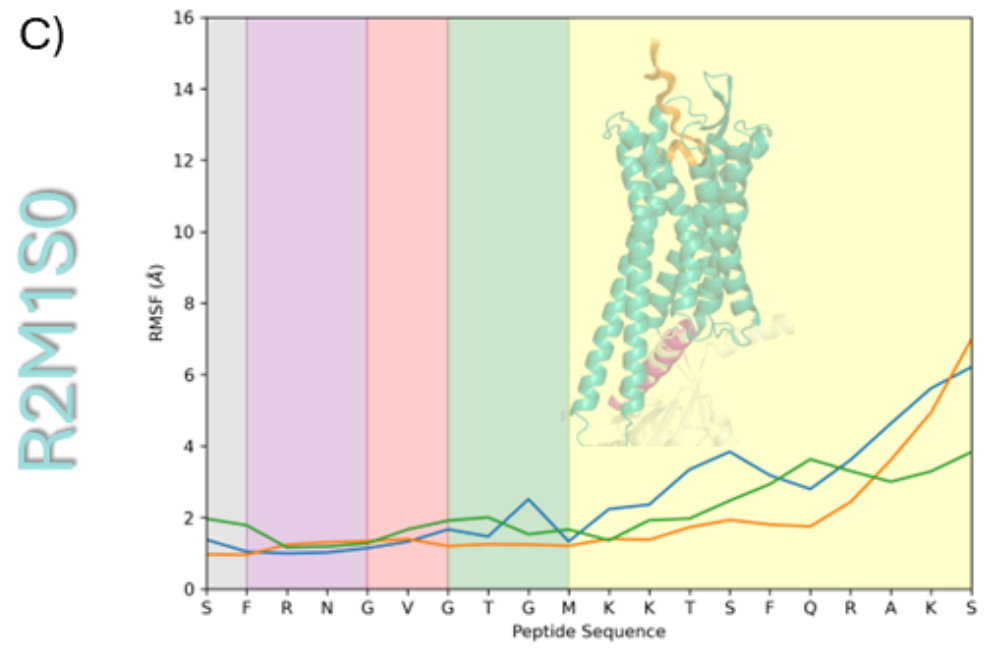

RMSD [Å]

	Cα Receptor	Cα NPS(1-10)
R1	2.26 ± 0.35	**2.08 ± 0.55**
R2	2.25 ± 0.32	2.37 ± 0.37
R3	**2.19 ± 0.30**	2.43 ± 0.46

D)

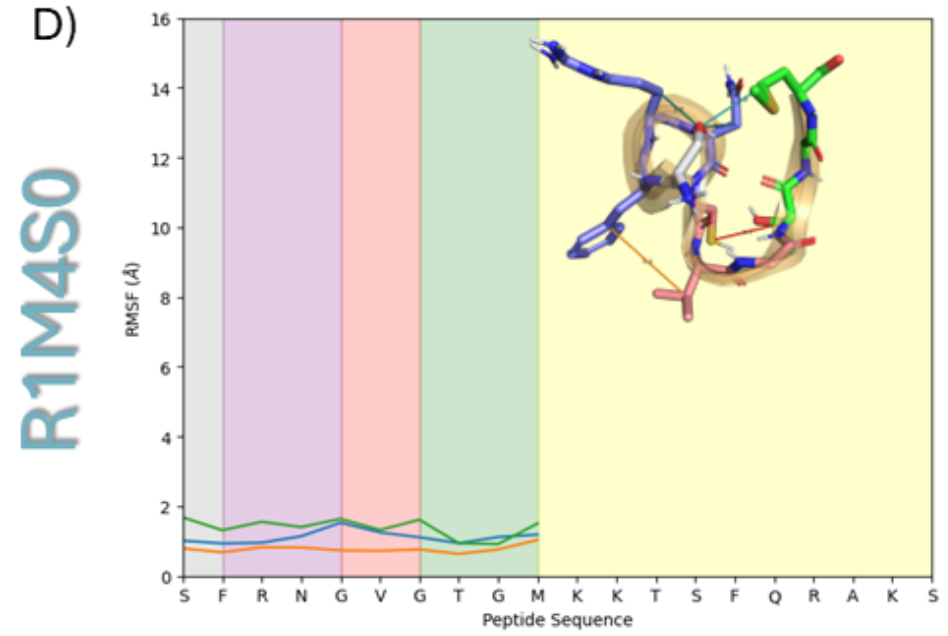

RMSD [Å]

	Cα Receptor	Cα NPS(1-10)
R1	**2.13 ± 0.33**	2.39 ± 0.28
R2	2.32 ± 0.29	**2.26 ± 0.22**
R3	2.13 ± 0.37	3.21 ± 0.57

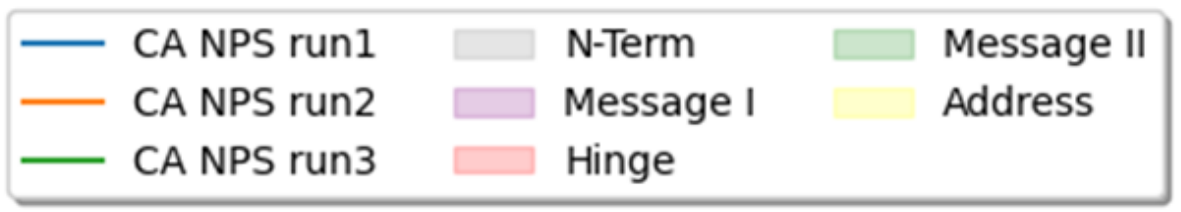

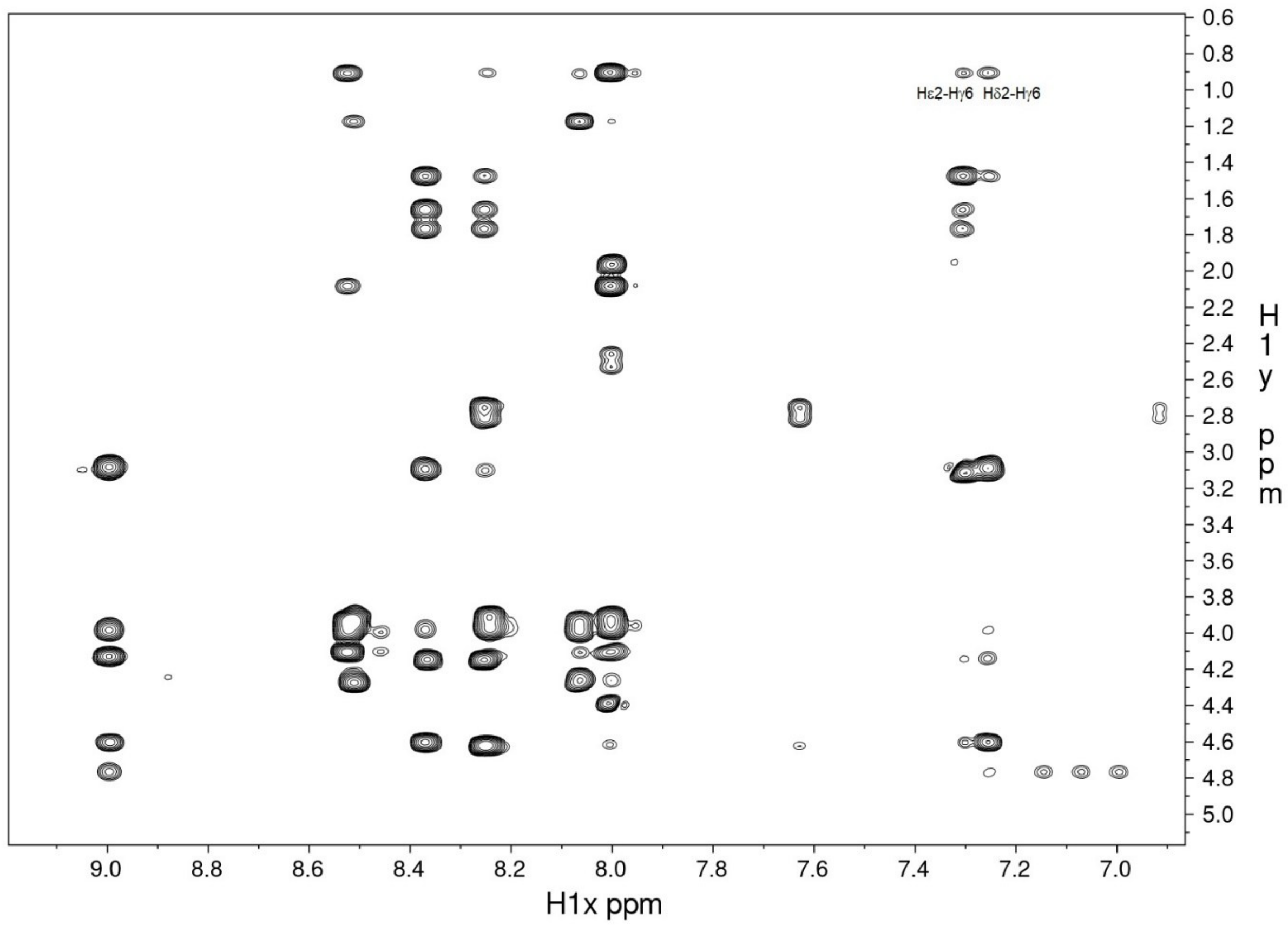

Figure S2. Selected region of the NOESY spectrum of hNPS(1-10). Important NOEs are labeled.

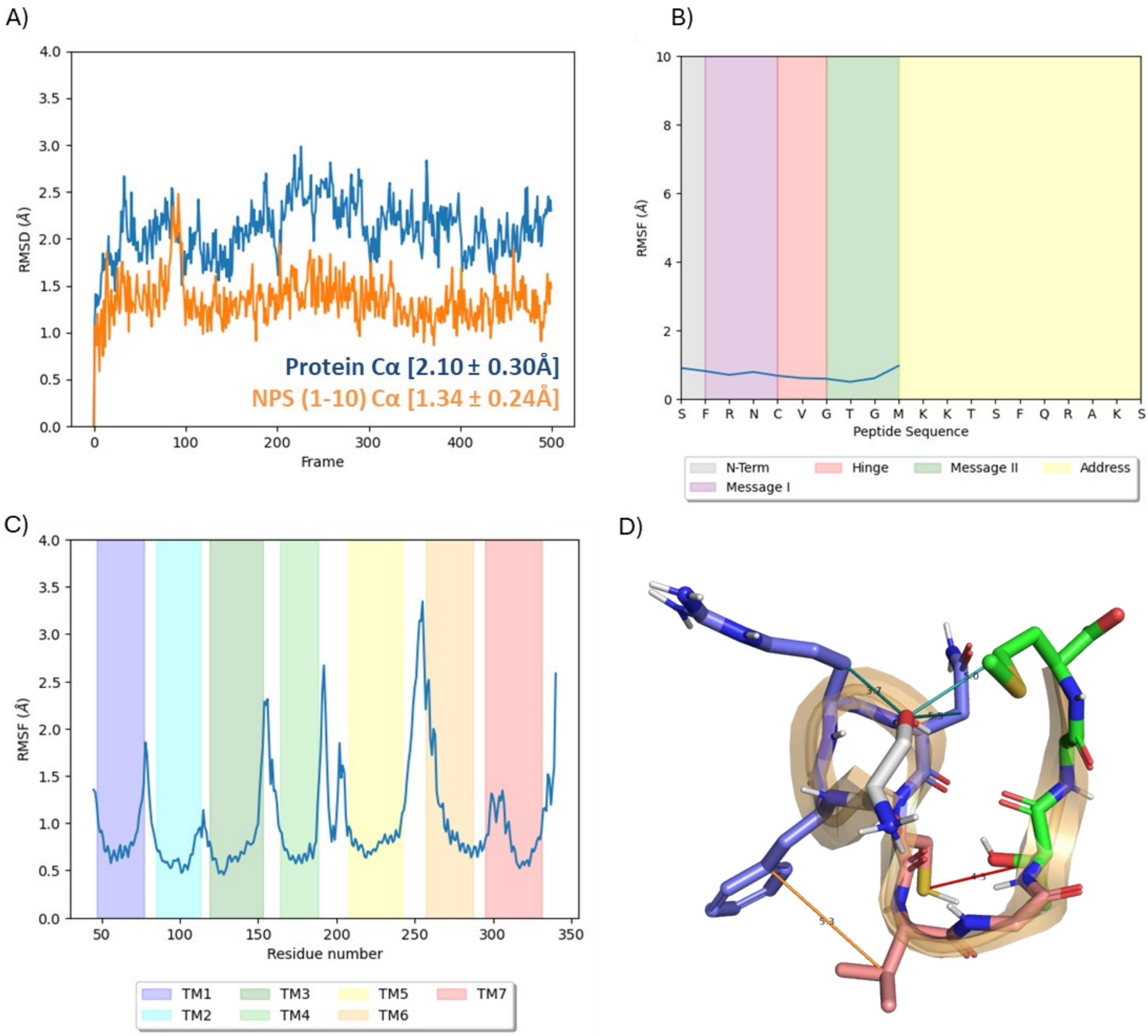

Figure S3. MD analysis of the [Cys^5]hNPS(1-10) system: A) RMSD profile of the receptor (blue) and peptide (orange) during the simulated time (50 ns); average B) Peptide and C) Receptor RMSF profile. D) Pseudo-cyclic conformation extracted from the trajectory, confirming the potential for cyclic analogue design.

HPLC traces of peptides **1**-**20**

Compound **1**

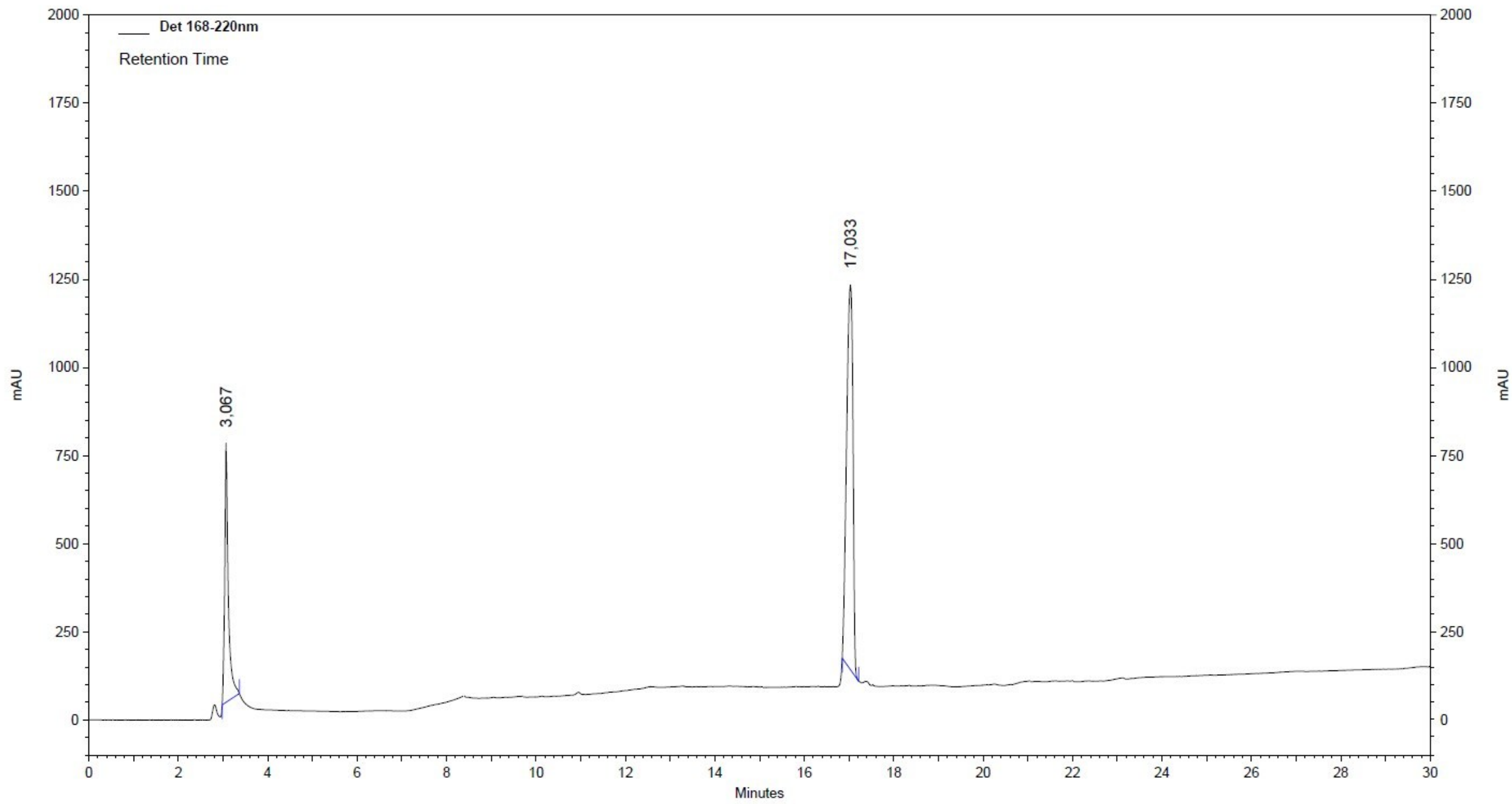

Compound **2**

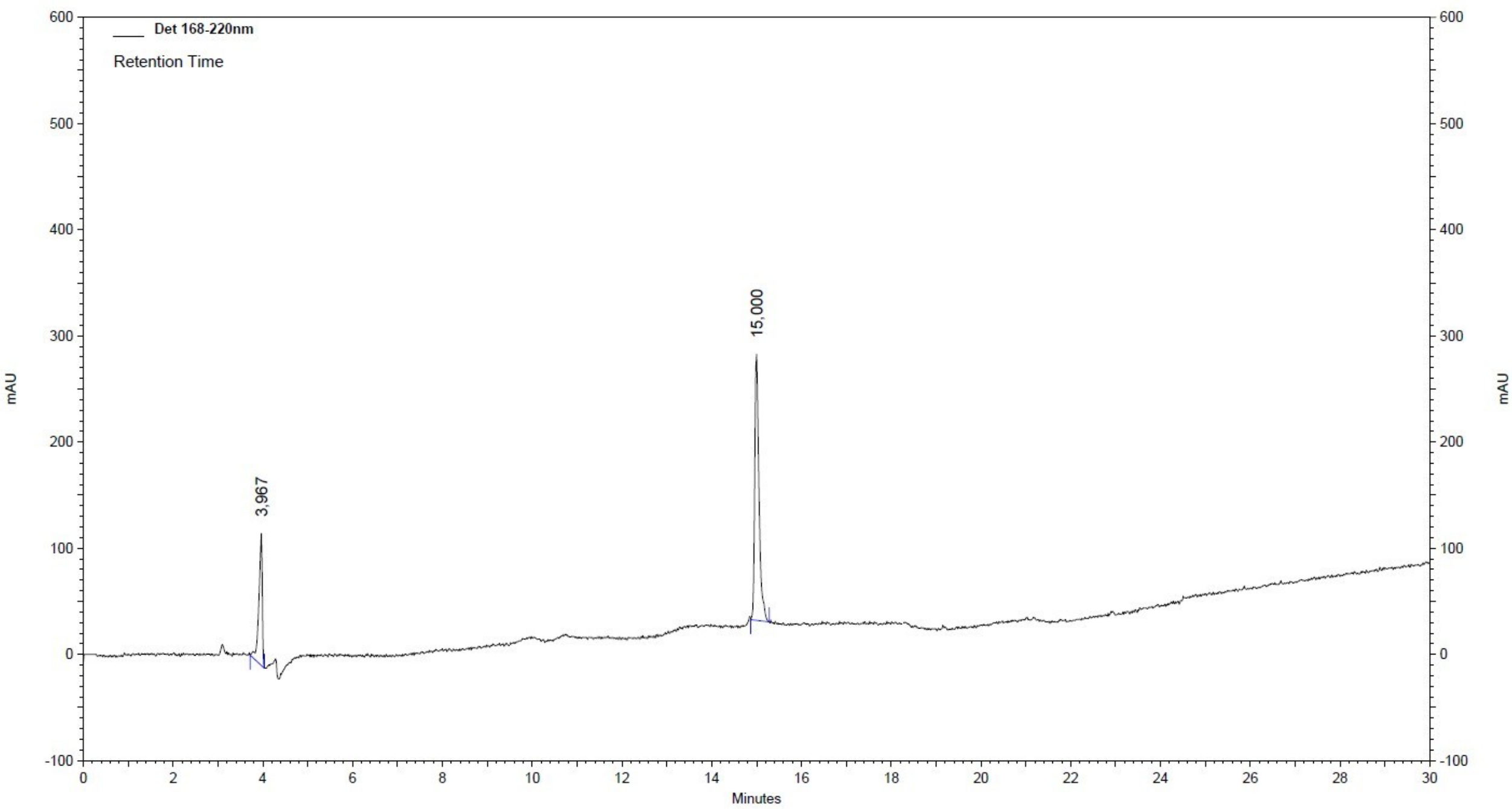

Compound **3**

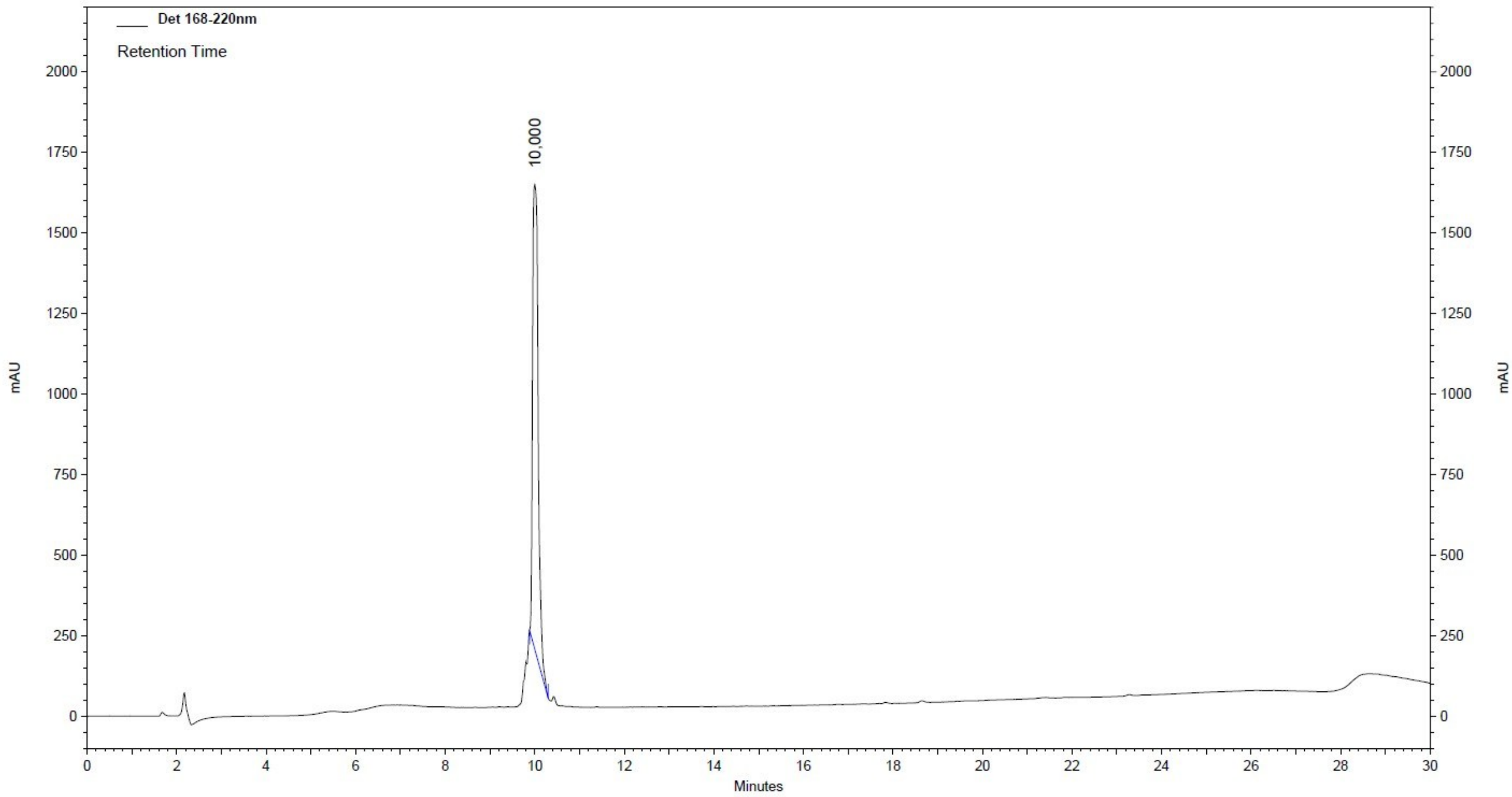

Compound **4**

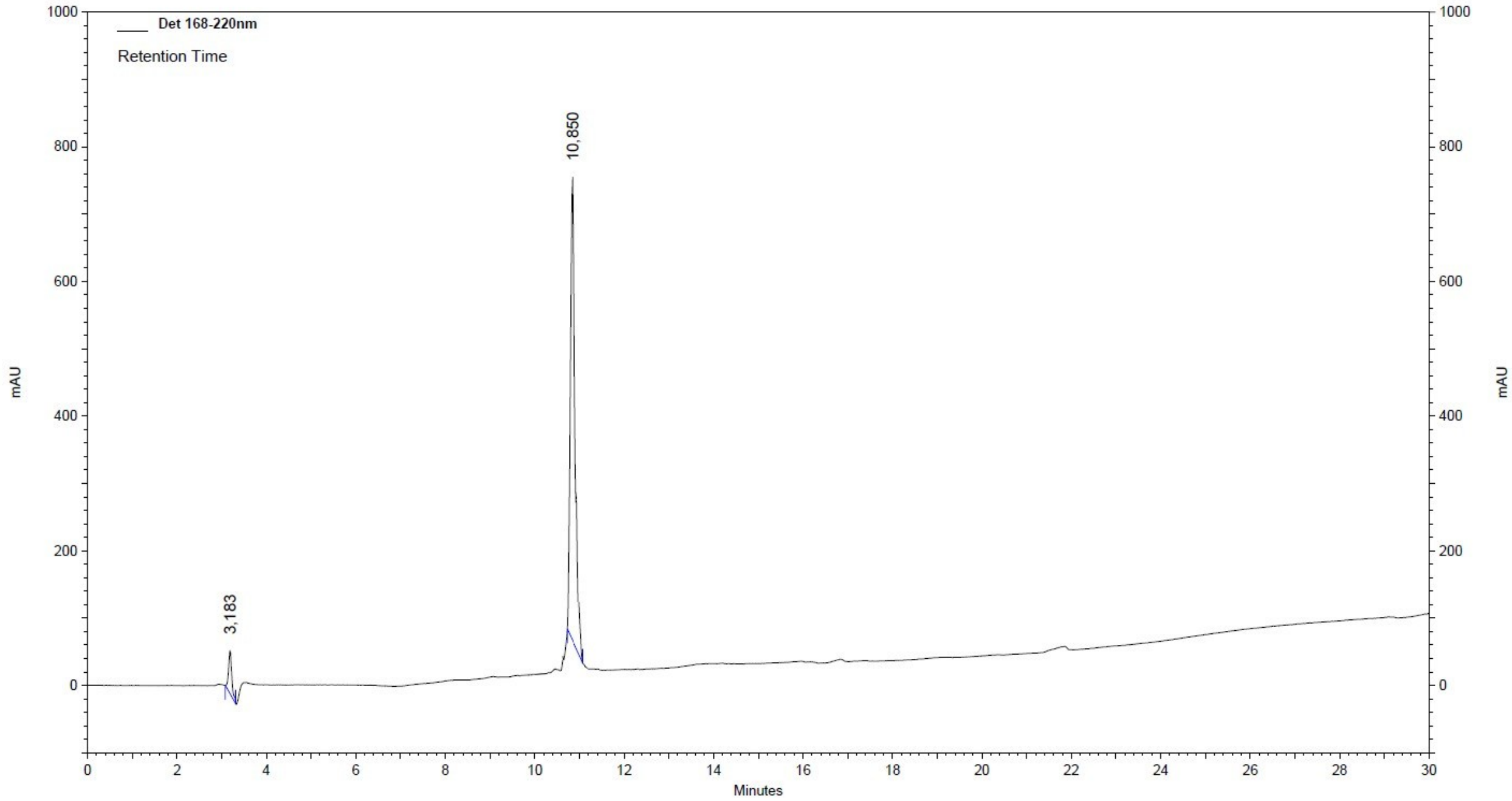

Compound **5**

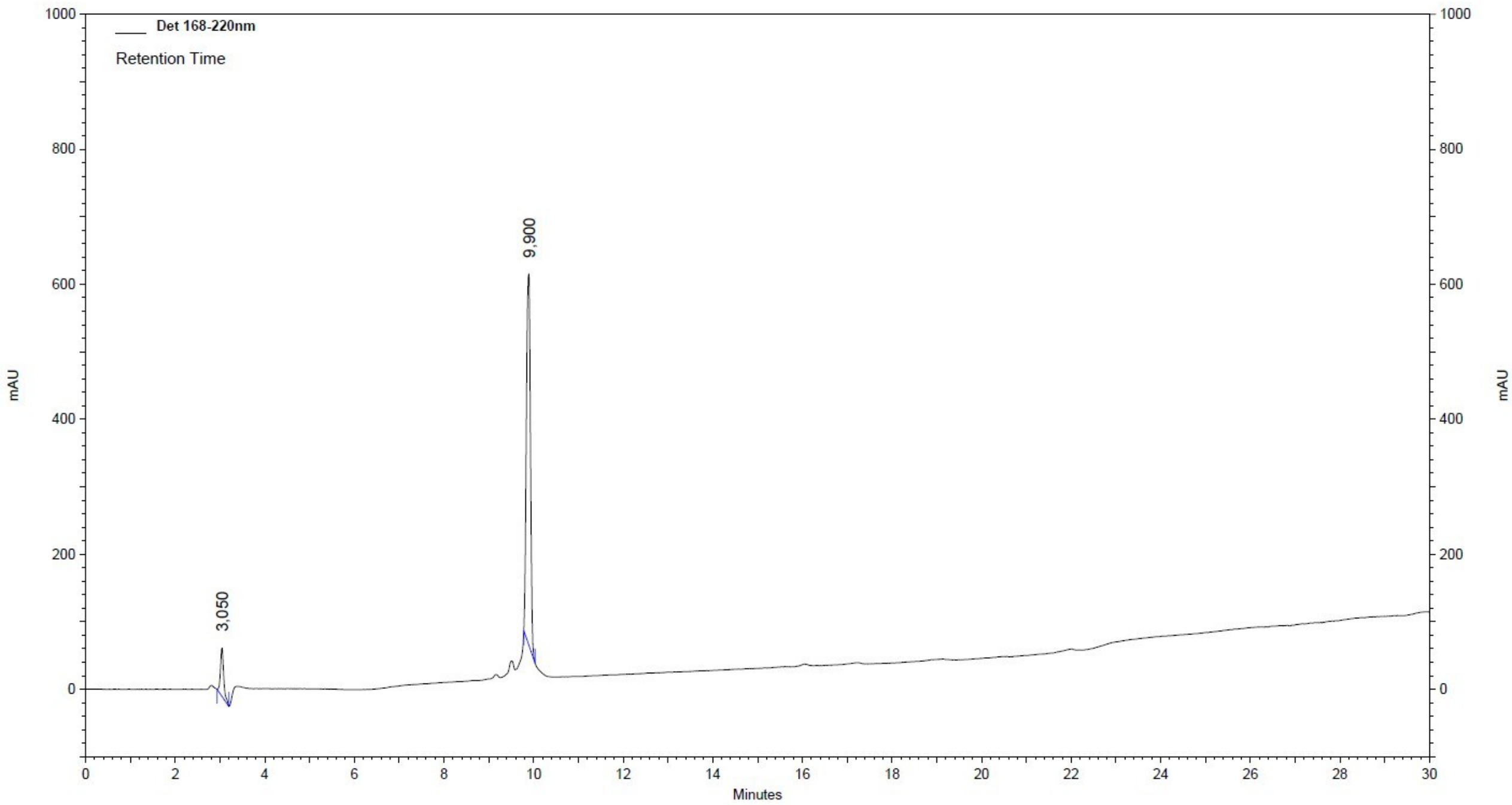

Compound **6**

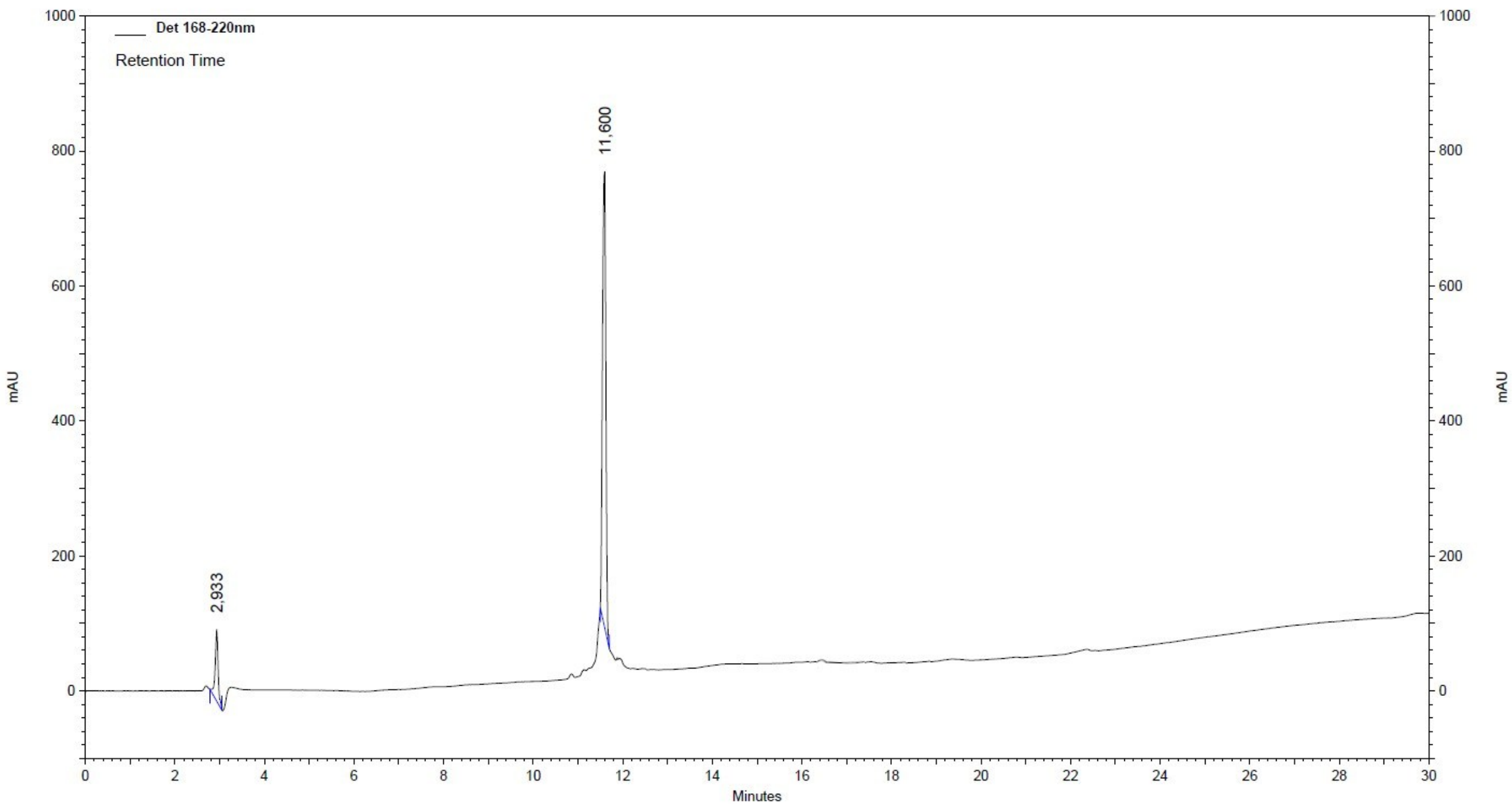

Compound **7**

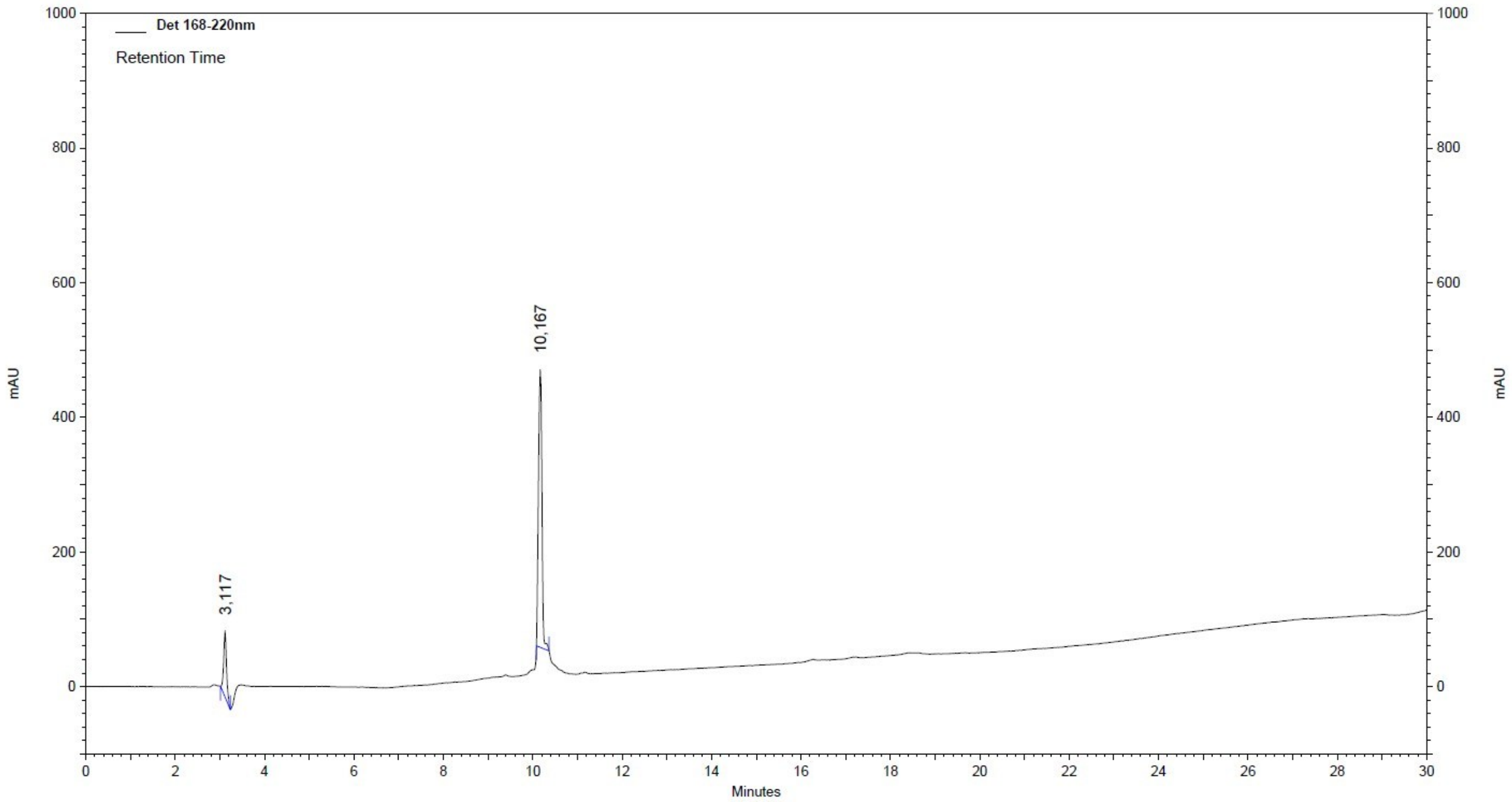

Compound **8**

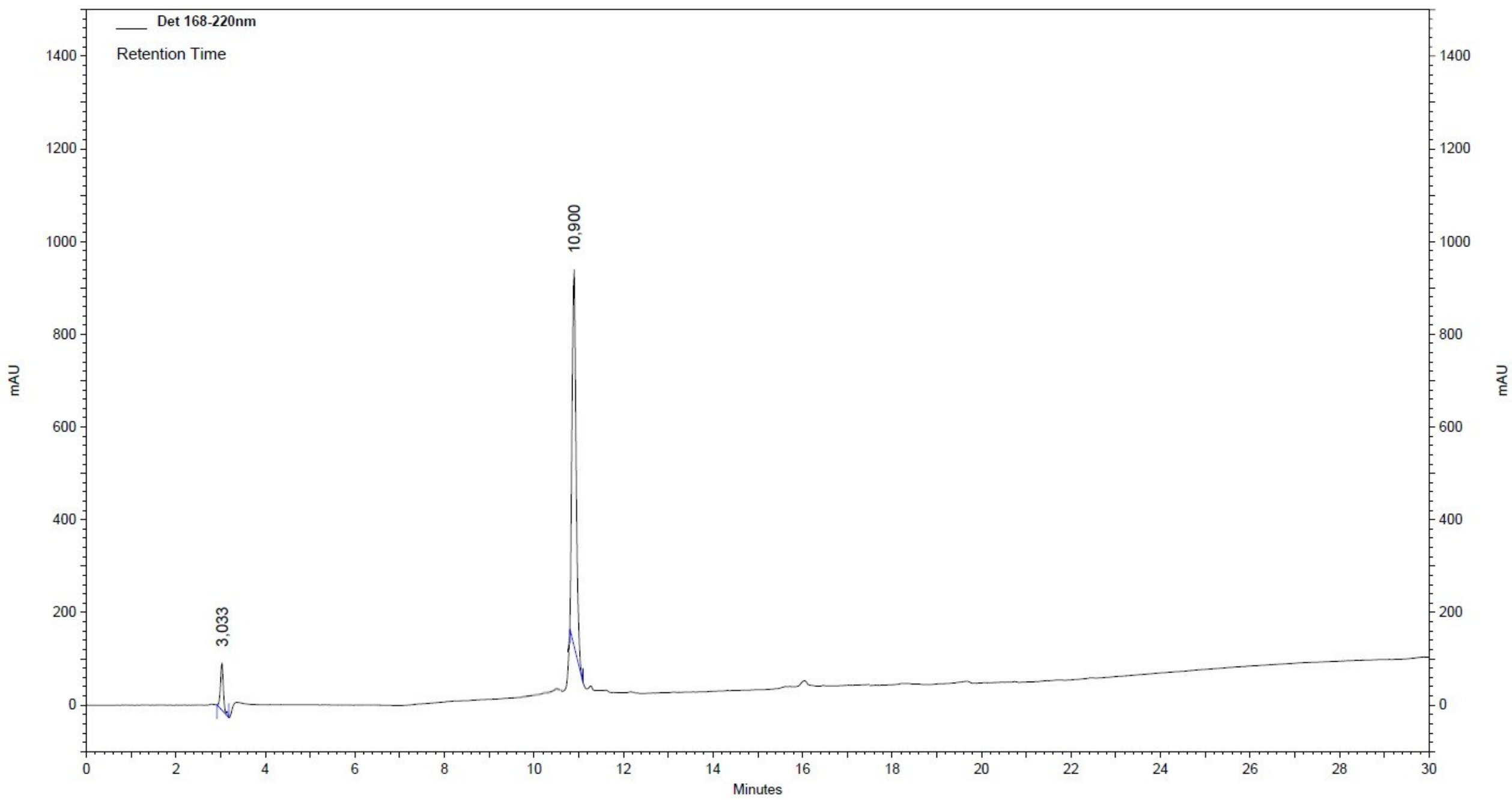

Compound **9**

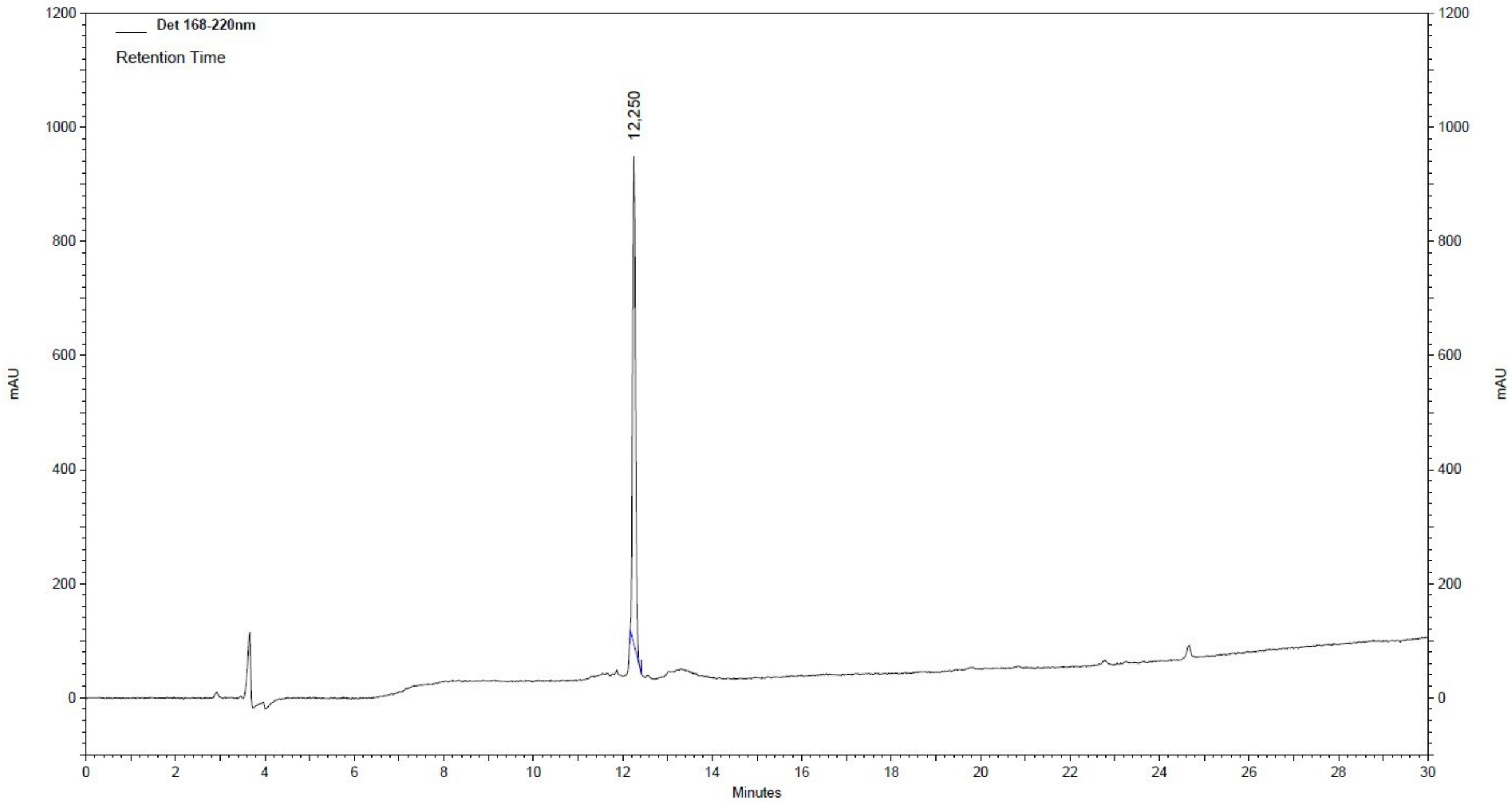

Compound **10**

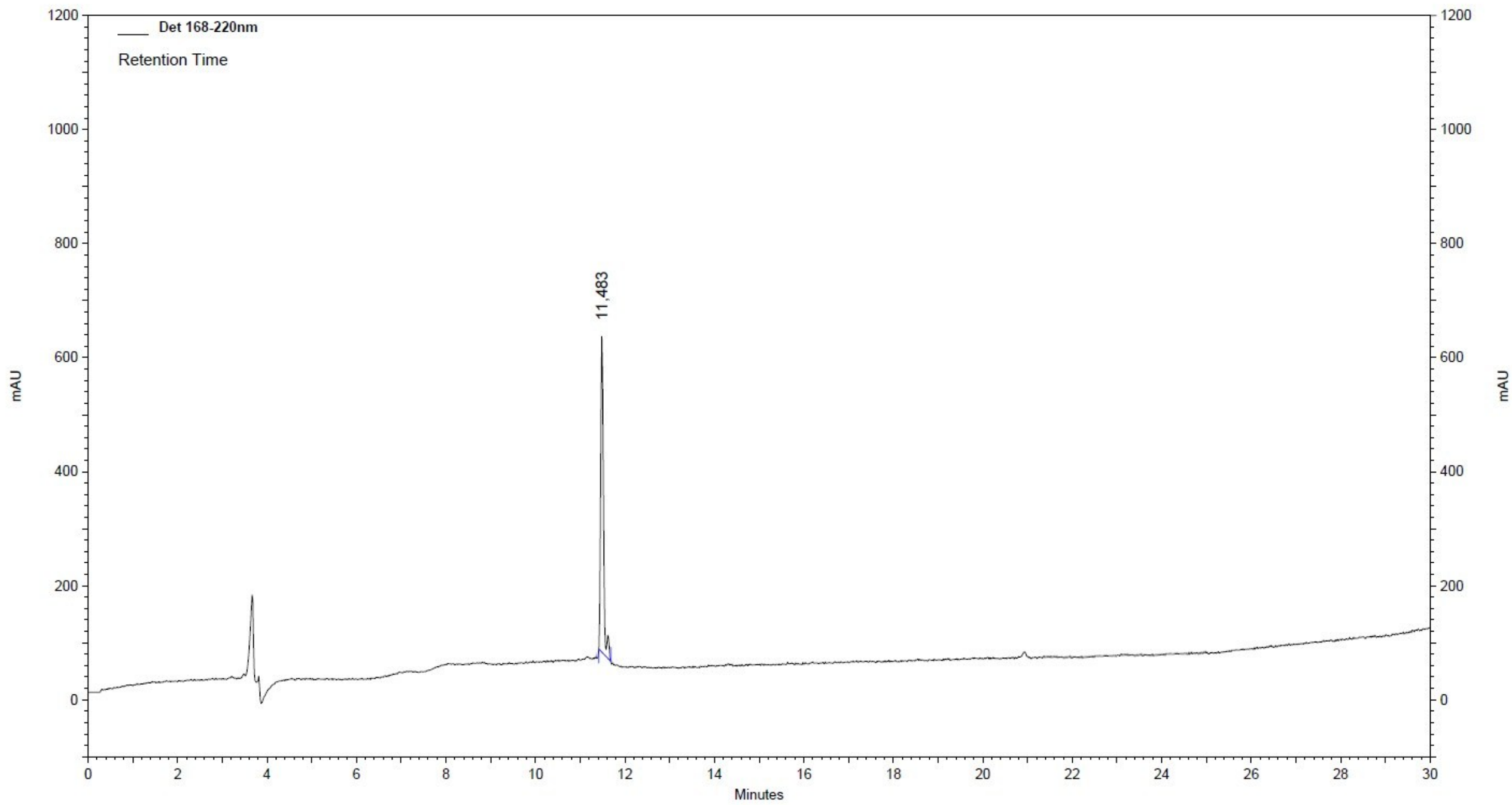

Compound **11**

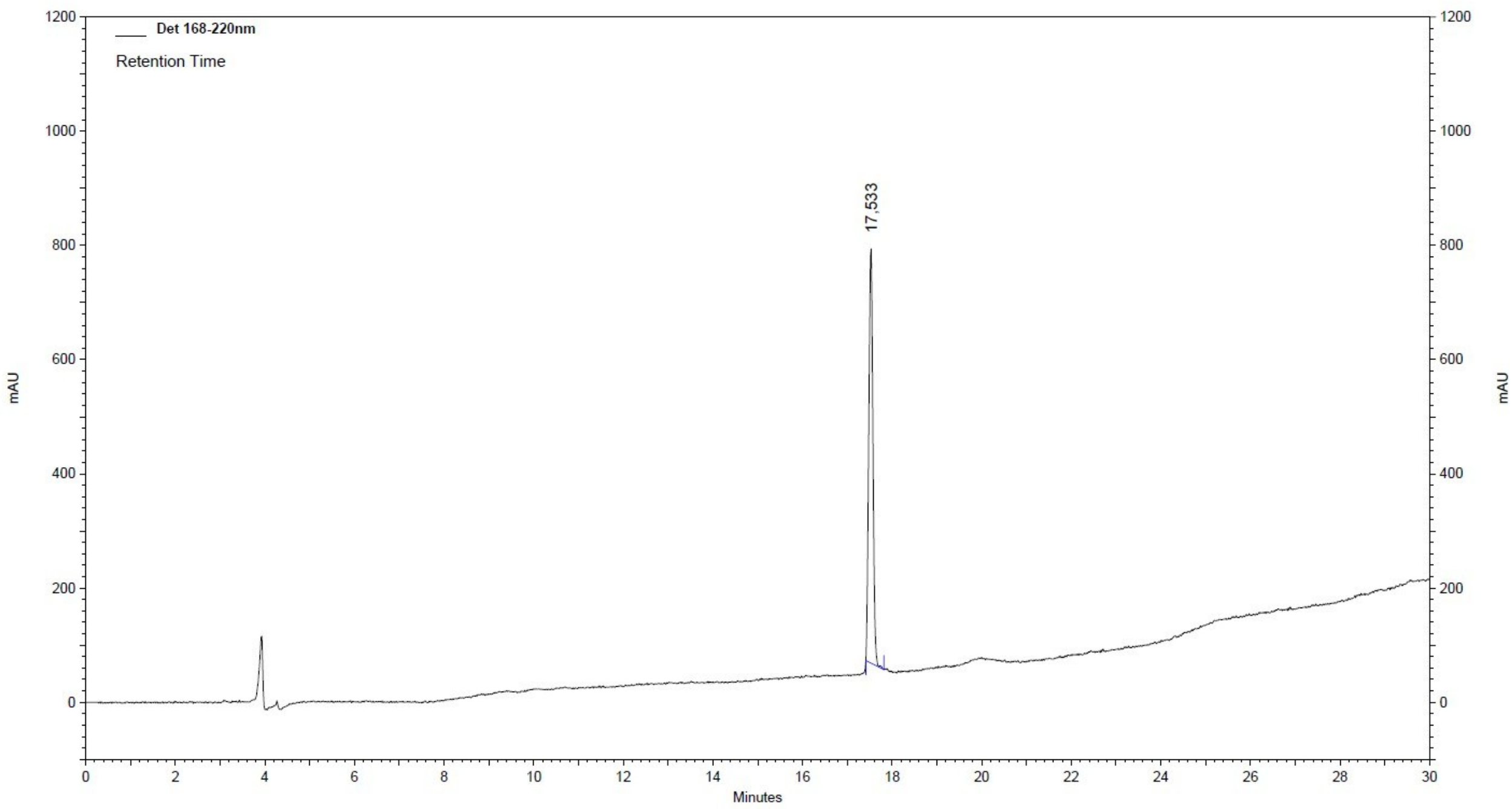

Compound **12**

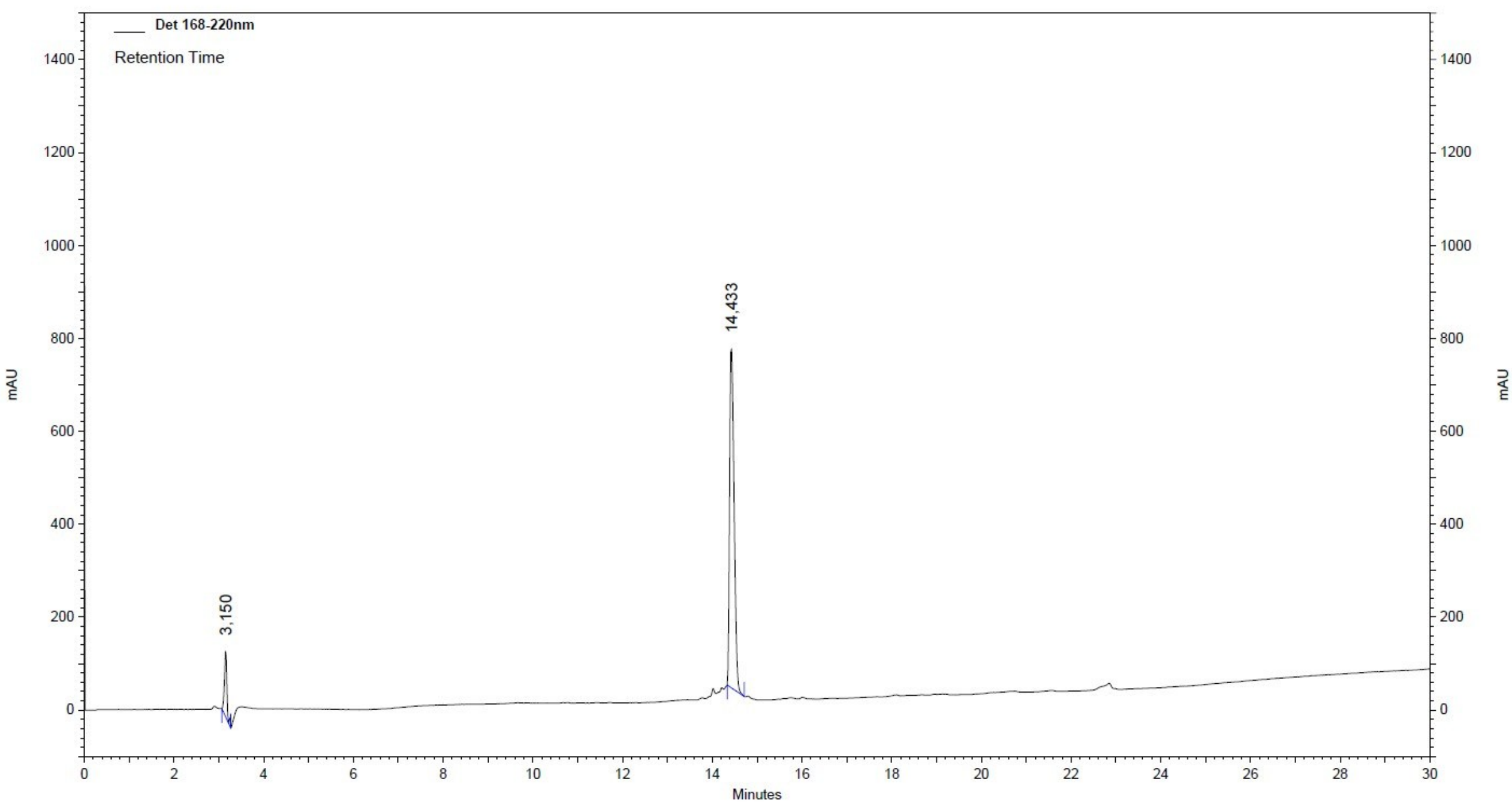

Compound **13**

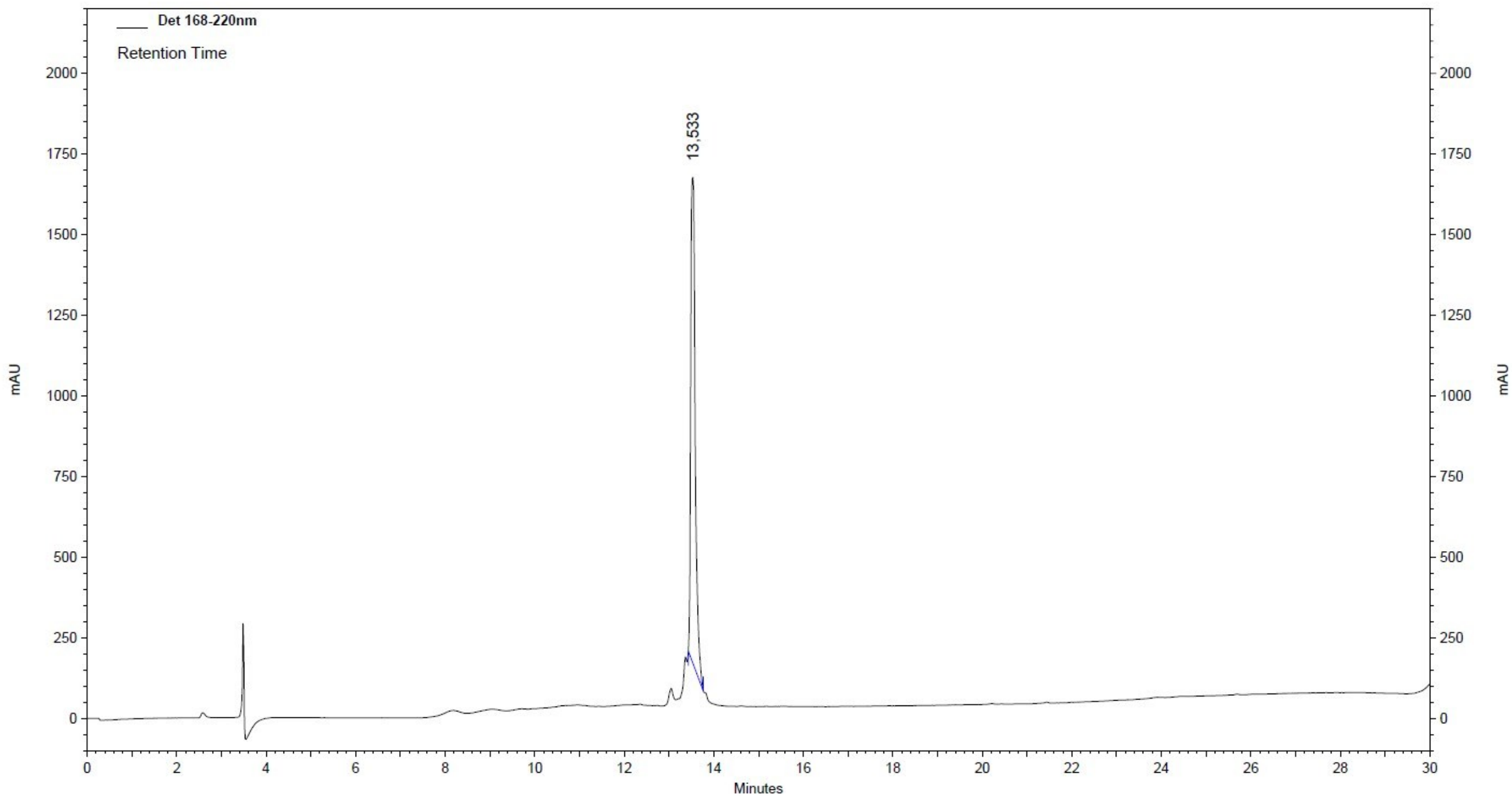

Compound **14**

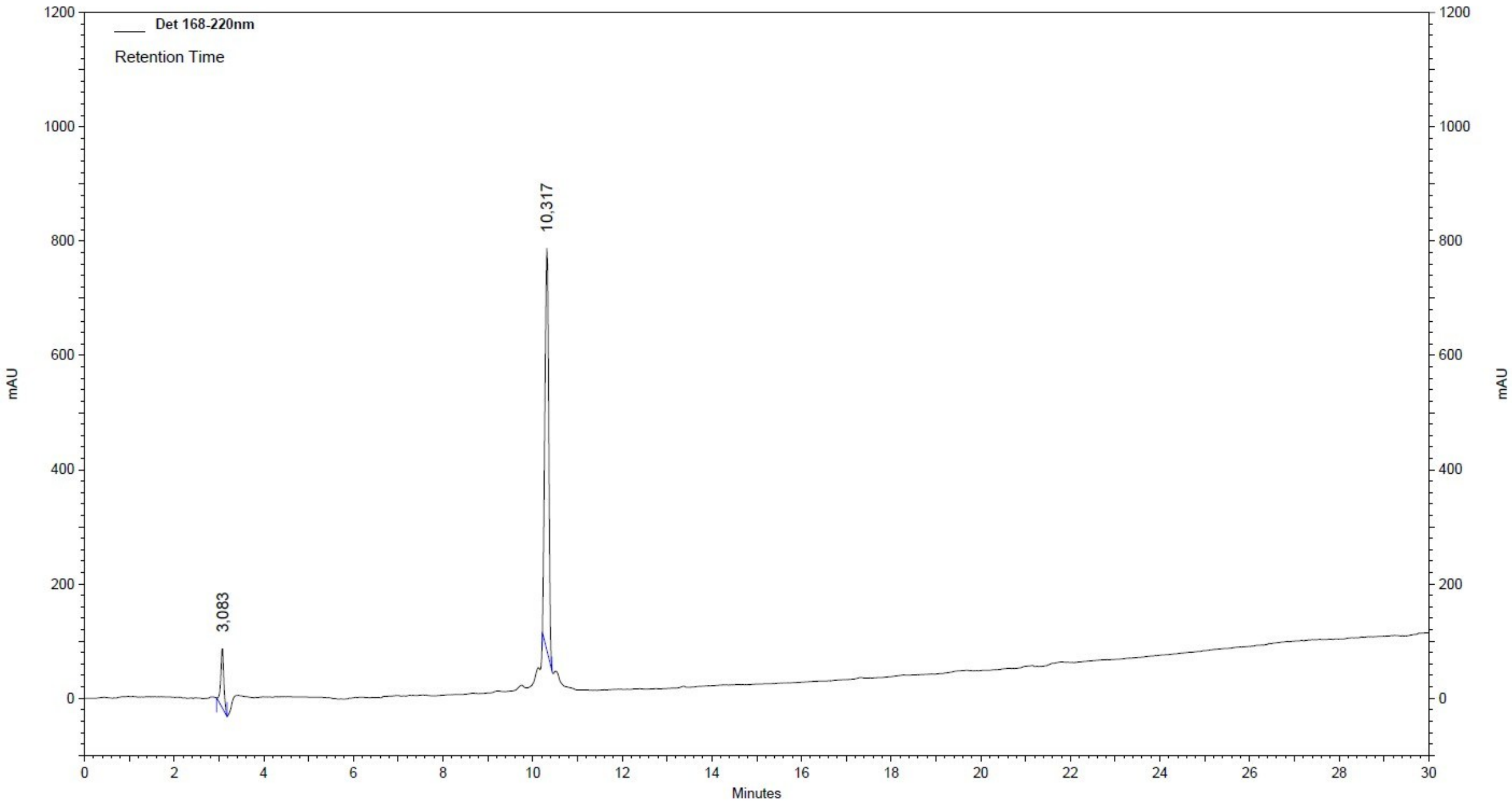

Compound **15**

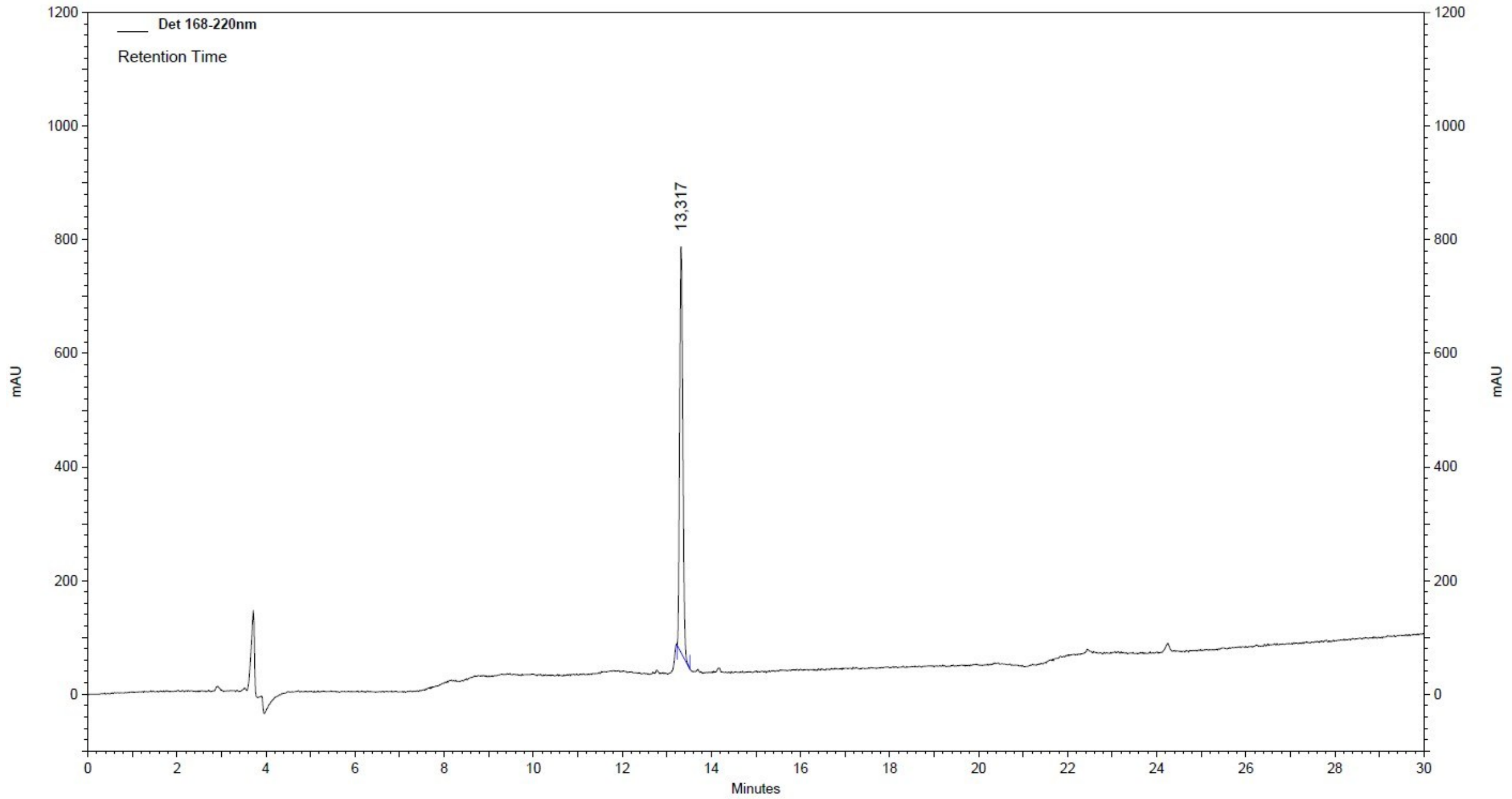

Compound **16**

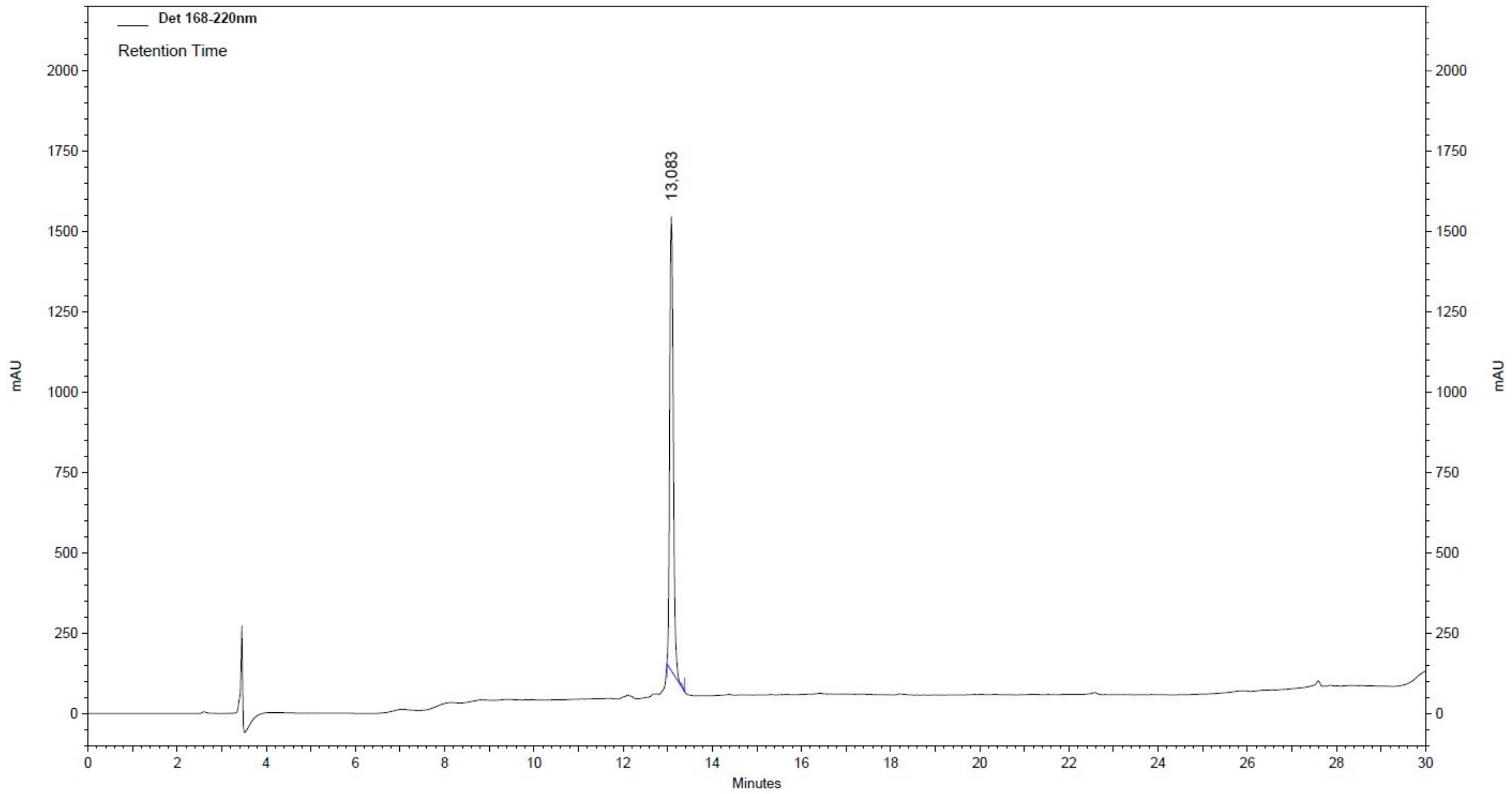

Compound **17**

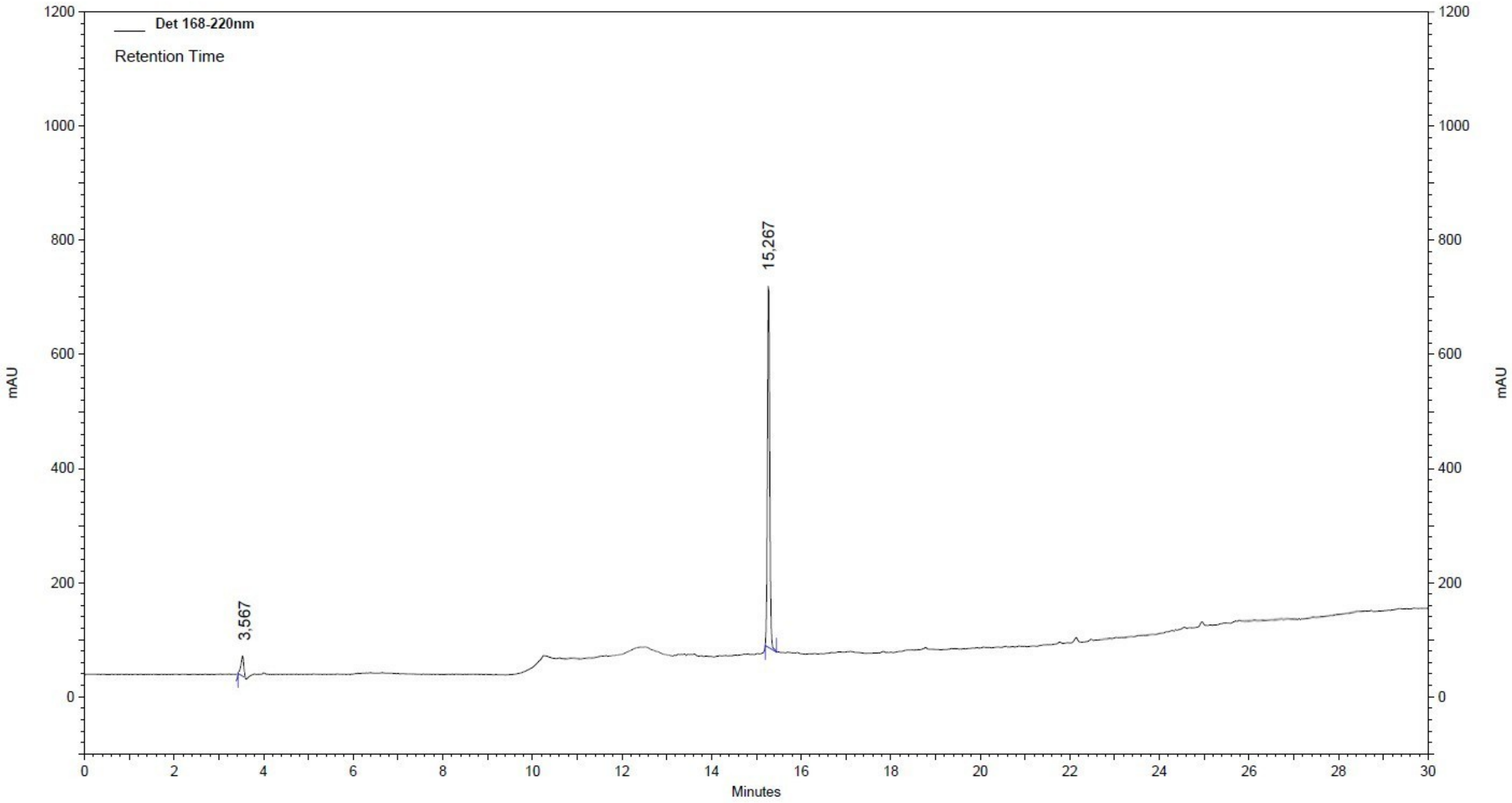

Compound **18**

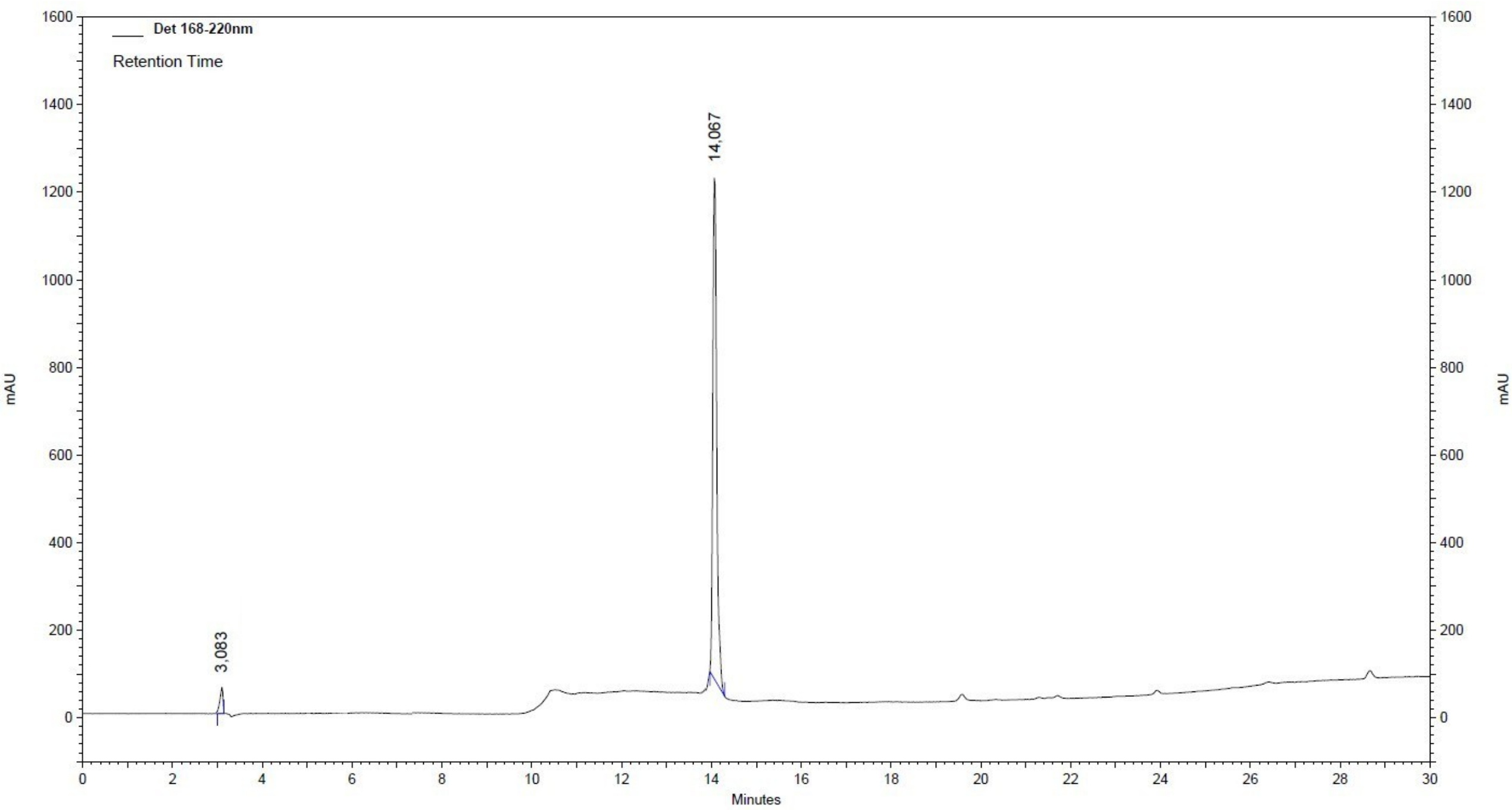

Compound **19**

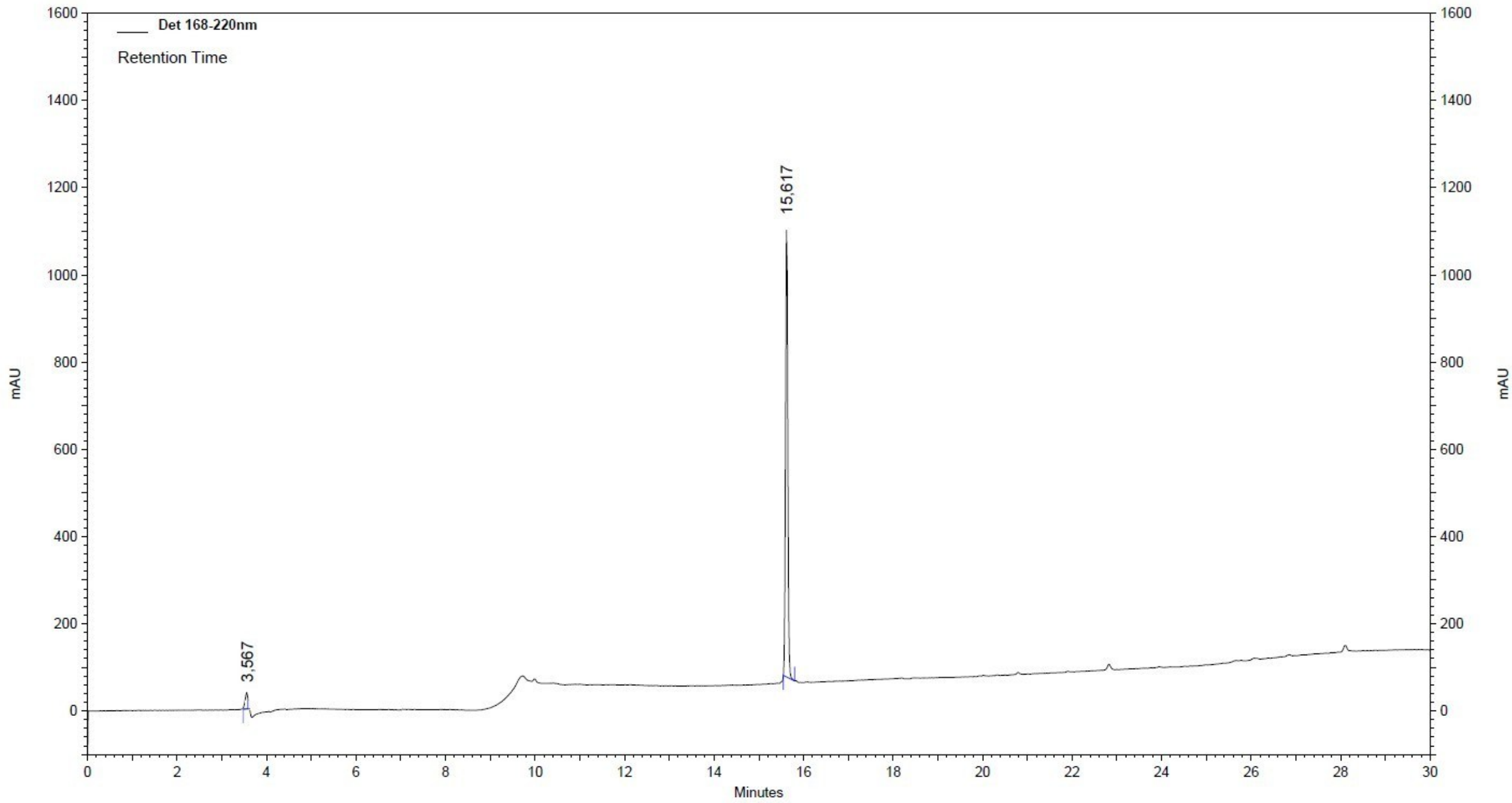

Compound **20**

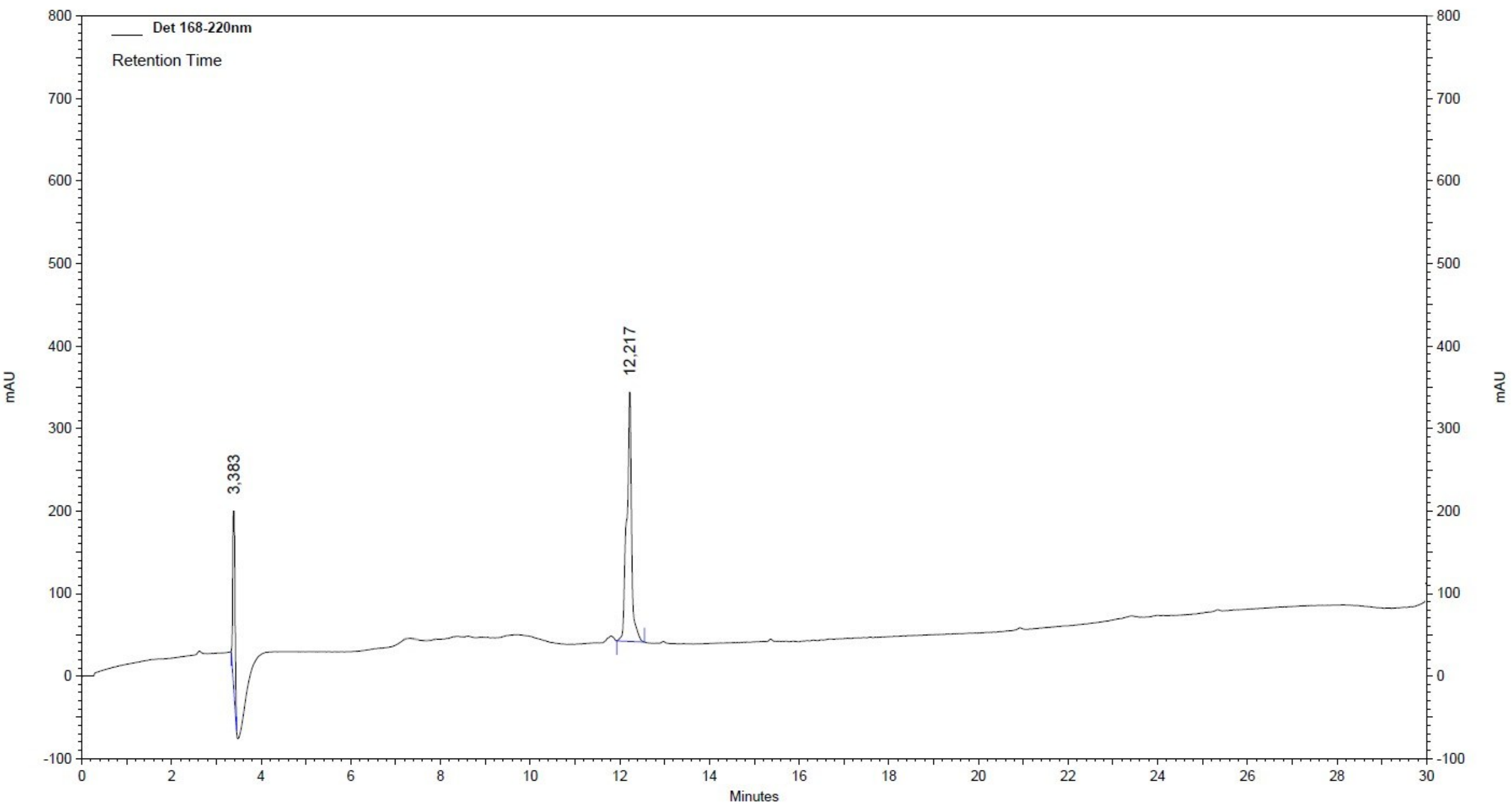

Table S3. ESI-MS data (calculated and observed) for the synthesized peptides.

Cpd	Formula	$[M+H]^+$ calcd	$[M+H]^+$ found
**1**	$C_{44}H_{68}N_{16}O_{12}S$	1044.52	1044.77
**2**	$C_{36}H_{62}N_{14}O_{13}S_3$	994.40	994.94
**3**	$C_{37}H_{64}N_{14}O_{13}S_3$	1008.42	1008.74
**4**	$C_{46}H_{71}N_{17}O_{13}S$	1101.54	1101.83
**5**	$C_{43}H_{65}N_{17}O_{13}S$	1059.49	1059.55
**6**	$C_{46}H_{71}N_{17}O_{13}S$	1101.54	1101.51
**7**	$C_{40}H_{67}N_{17}O_{14}S$	1041.50	1041.40
**8**	$C_{40}H_{67}N_{17}O_{14}S$	1041.50	1041.65
**9**	$C_{37}H_{64}N_{14}O_{14}S_3$	1024.41	1025.01
**10**	$C_{39}H_{68}N_{14}O_{14}S_3$	1052.44	1052.79
**11**	$C_{40}H_{70}N_{14}O_{14}S_3$	1066.46	1066.77
**12**	$C_{37}H_{61}N_{17}O_{14}S$	999.45	999.54
**13**	$C_{38}H_{66}N_{14}O_{14}S_3$	1038.43	1038.44
**14**	$C_{40}H_{67}N_{17}O_{14}S$	1041.50	1041.67
**15**	$C_{42}H_{66}N_{14}O_{13}S_3$	1070.43	1070.79
**16**	$C_{44}H_{70}N_{14}O_{13}S_3$	1098.46	1099.06
**17**	$C_{41}H_{72}N_{14}O_{14}S_3$	1080.47	541.0 ($[M+2H]^{2+}$ found)
**18**	$C_{38}H_{66}N_{14}O_{14}S_3$	1039.23	1039.60
**19**	$C_{40}H_{67}N_{17}O_{14}S$	1041.50	1040.41
**20**	$C_{37}H_{61}N_{17}O_{14}S$	1000.08	1000.00
